# A Systematic Review on the Influence of Feeding Expressed Mother’s Own Milk Using Varying Expression Practices or Treatments on Health and Growth of Recipient Infants

**DOI:** 10.1016/j.advnut.2025.100523

**Published:** 2025-09-19

**Authors:** Serena Gandino, Tanya Cassidy, Marzia Giribaldi, Magdalena Babiszewska-Aksami, Agnieszka Bzikowska-Jura, Laura Cavallarin, Karolina Karcz, Daniel Klotz, Chiara Peila, Carolyn Smith, Bartłomiej Walczak, Aleksandra Wesolowska

**Affiliations:** 1 Nuffield Department of Women's & Reproductive Health, University of Oxford, Oxford, United Kingdom; 2 Neonatal Unit, Department of Public Health and Pediatrics, University of Turin, Turin, Italy; 3 Kathleen Lonsdale Institute for Human Health Research, Maynooth University, Maynooth, Ireland; 4 Institute of the Sciences of Food Production, National Research Council, Turin, Italy; 5 Human Milk Bank Foundation, Warsaw, Poland; 6 Laboratory of Human Milk and Lactation Research, Department of Medical Biology, Medical University of Warsaw, Warsaw, Poland; 7 Department of Neonatology, Wroclaw Medical University, Wroclaw, Poland; 8 Bethel Center of Pediatrics, Department for Neonatology and Pediatric Intensive Care Medicine, University Hospital OstWestfalenLippe (OWL) of Bielefeld University, Nordrhein-Westfalen, Bielefeld, Germany; 9i Bodleian Health Care Libraries, University of Oxford, Oxford, United Kingdom; 10 Institute of Applied Social Sciences, University of Warsaw, Warsaw, Poland

**Keywords:** mother’s own milk, hand expression, pasteurization, freezing, hindmilk, infant growth, CMV

## Abstract

When feeding at the breast is not possible, infants can still receive expressed mother’s own milk (MOM). Method of expression, hygiene practices and settings during expression, and processing can affect MOM composition. This study aimed to review current evidence on the influence of feeding MOM expressed using varying expression methods, hygiene practices or settings during expression, or treatments on the health and growth of recipient infants. We systematically searched CENTRAL, CINAHL, clinicaltrials.gov, Embase, Emcare, EU trials, Global Health, Global Index Medicus, MEDLINE, Scopus, Web of Science, and WHO for primary research studies, including observational studies, published up to March 2024 evaluating different methods of MOM expression, hygiene practices or settings during expression, and methods processing of MOM and reporting clinical outcomes on recipient infants. Key outcomes of interest were growth, mortality, morbidity, feeding tolerance, adverse events, cytomegalovirus (CMV) infection, retroviral infection, other infections, nutrient deficiencies, neurodevelopment, and breastfeeding. Qualitative thematic synthesis was conducted. An evidence gap map was produced using the Grading of Recommendations, Assessment, Development, and Evaluation approach. Of 29,320 studies screened, 45 met the inclusion criteria. No expression method or pump type showed clear benefits for breastfeeding rates or infant growth. Three studies reported improved weight gain in infants receiving hindmilk. Evidence on the effect of processing methods on morbidity and mortality was inconclusive. Limited evidence was found on the efficacy of the freeze-thaw cycle in reducing CMV transmission, whereas pasteurization proved more effective. No studies assessed clinical outcomes related to hygiene practices or expression settings. The use of hindmilk improves infant weight gain with some certainty of evidence. Hand expression of MOM has similar efficacy to that of electric pumping on the growth of recipient infants, including preterm infants. Evidence on clinical outcomes of different MOM expression practices and treatments is very limited. This work underscores the need for future studies to address the substantial evidence gaps identified.

This study was registered at PROSPERO as CRD42024523299.

**Statement of Significance**

Previous reviews have focused on the effects of handling practices for expressed mother’s own milk (MOM), such as expression methods, hygiene practices/settings during expression, and storage and processing treatments, on the composition of MOM; however, changes in composition do not necessarily correspond to changes in clinical outcomes. To our knowledge, this is the only systematic review to evaluate the effects of these practices on a large number of clinically relevant outcomes (growth, main morbidities, feeding tolerance, adverse events, viral and retroviral infections, other infections, nutrient deficiencies, neurodevelopment, and breastfeeding) of recipient infants. In this context, the present work is important to identify gaps in knowledge, including methodological limitations of existing evidence, and guide future research.

## Introduction

Breastfeeding is the biological norm for infants [1]. In certain scenarios, feeding at the breast is not possible because of either neonatal or maternal reasons. In such instances, infants can still be fed their mothers’ own milk (MOM) expressed and provided to the infant via a bottle, cup, or similar vessel. Milk expression enables infants who cannot feed directly at the breast to still receive the beneficial properties of MOM. In some specific cases, heat treatments can be applied to MOM to reduce risk of viral infection [human T-cell leukemia/lymphoma virus or human T-lymphotropic virus (HTLV), cytomegalovirus (CMV), and varicella zoster virus] [2]. This is particularly relevant in the case of very low birth weight and very preterm infants to reduce risk of transmitting CMV, which can cause severe sepsis in this vulnerable population [[3], [4], [5]]. Moreover, in case of HIV-positive mothers, studies have evaluated heat treatments of MOM to reduce risk of HIV transmission [6].

The way MOM is expressed, the hygiene practices applied during expression, and the setting where expression occurs, as well as the processing conditions applied to MOM may influence milk composition and may potentially impact the health and growth outcomes of recipient infants (Table 1) [5,[7], [8], [9]].

**TABLE 1:** Description of the interventions that may influence the health and growth outcomes of recipient infants that have been overlooked in the present systematic review.

Factors	Interventions
Methods of milk expression	There is a wide variety of methods to express milk. The one which is universally available is hand expression, in which milk ejection is stimulated by hand compression of the breast. Another option would be the hot jar method, in which a glass jar, warmed up with hot water, is cooled at the base with a cold cloth: the temperature gradient creates a vacuum effect, which promotes milk ejection. Milk can also be expressed through a pump, which creates a negative pressure that stimulates milk flow. Pumps can be manual, battery-powered, or electric. Available evidence on the effectiveness, safety, and effect on milk composition shows no difference regarding the contamination of human milk across the different methods of expression [7]. Regarding the effect on milk composition, milk expressed via hand expression or via a large electric pump was found to have a higher protein content, which could be beneficial for promoting infants’ growth, especially those born preterm. Hand expression was also associated with higher sodium content, which could contribute to the recovery of the sodium deficiency that typically affects preterm infants, impairing their growth. Fat content was higher when expression was accompanied by breast massage. Milk energy content was similar across the different methods of expression [7].
Hygiene practices and settings during milk expression	Human milk can get contaminated with exogenous bacteria during expression and handling. These bacteria may potentially cause sepsis in recipient infants, especially those born prematurely. Hence, appropriate hygiene practices need to be applied to ensure milk safety [5]. Mothers’ hand hygiene can be achieved by washing hands with soapy water or with an alcohol-based sanitizer. Breast cleansing can be performed with water only, water and soap, or antibacterial wipes. Disinfectants can be used to clean the pumping area. When using a pump, parts of the equipment that come into contact with the breast can be rinsed before expressing; after pumping, the kit can be cleaned and disinfected either by washing by hand with warm soapy water, by using a dishwasher, or by boiling the equipment. Also, the setting of expression of MOM might have an influence, as some reports have revealed higher contamination in samples expressed at home than in those expressed in the hospital [8].
Processing of expressed milk	Human milk is not sterile but contains a number of microorganisms, the so-called “human milk microbiota,” that play an essential role in shaping the infant microbiome. It may also serve as a vector for infectious diseases and, therefore, might be subjected to different kinds of heat treatments to reduce the risk of transmission. Risks of transmission must be outweighed by the deleterious effect of any kind of treatment on the immunological and nutritional properties of MOM. Cold storage at different temperatures and for different durations has been used traditionally for MOM in order to both extend its duration and decrease/eliminate specific viral pathogens. Low-temperature storage is also effective in preventing bacterial proliferation, although it may negatively affect the immunological and nutritional properties of human milk. Alternatively, MOM can be pasteurized. Among the techniques, Holder pasteurization represents the best compromise currently available, offering safety but impairing milk quality, which may influence health and growth outcomes of recipient infants. Other technologies have been evaluated, aiming to provide microbial safety while maintaining human milk quality to the utmost degree possible [9].

Abbreviation: MOM, mother’s own milk.

Previous reviews have focused on the effects of those interventions on the composition of MOM. However, changes in composition do not necessarily correspond to changes in clinical outcomes. To our knowledge, no systematic review has evaluated the effects of different methods of MOM expression, hygiene practices or settings during expression, or methods of processing of MOM on the clinical outcomes of recipient infants.

In this review, we examined the impact of feeding expressed MOM collected using varying expression methods, hygiene practices or settings during expression, or treatments on the health and growth outcomes of recipient infants.

## Methods

### Search strategy and selection criteria

We conducted a systematic review including heterogeneous trials in line with PRISMA guidelines [10].

Published or unpublished primary research studies were eligible for inclusion, without restrictions on type and including observational studies. We did not apply any setting, time frame, or language restriction, provided that the abstract was available in English. Translations of non-English manuscripts were performed using a large language model (DeepL Translate, Deepl SE). We excluded animal studies, conference proceedings, and papers with no original data. If eligible studies had unpublished or partially published results, we contacted the corresponding authors by e-mail to integrate available information with extracted data.

The population of interest was represented by term and preterm infants receiving expressed MOM, either alone or in combination with breastfeeding, donor human milk, or formula. The interventions of interest were expression methods, hygiene practices and settings, and processing applied to MOM. Health outcomes of interest included growth outcomes, mortality, morbidity [which comprised bronchopulmonary dysplasia (BPD), necrotizing enterocolitis (NEC), retinopathy of prematurity (ROP), intraventricular hemorrhage (IVH), and periventricular leukomalacia (PVL)], feeding tolerance (defined by duration of parenteral nutrition, time to full enteral feeding, or predefined feeding intolerance score), adverse events (defined as an undesired effect of the intervention under evaluation), CMV infection, retroviral infection, other infections (i.e., bacterial infections, fungal infections, viral infections other than CMV, and retroviruses), nutrient deficiencies, neurodevelopment (assessed through a standardized neurodevelopment assessment), breastfeeding rate, length of hospital stay, and other clinical outcomes.

We searched CENTRAL, CINAHL, clinicaltrials.gov, Embase, Emcare, EU trials, Global Health, Global Index Medicus, MEDLINE, Scopus, Web of Science, and WHO from database inception up to 12 March, 2024. The search terms included “mother’s own milk,” “expressed or stored,” and “effect on infants” [11]. The complete search strategy for each database is reported in the Supplemental Table 1, including the number of retrieved results.

We searched the reference lists of relevant reviews to identify additional studies. Moreover, we searched PubMed and Google Scholar for cited-by and similar articles (≤200 references for each list) of the 10 most cited eligible articles identified from the database search [12].

Using Covidence, pairs of reviewers independently screened titles and abstracts of all citations and full texts of selected references. Pairs of reviewers independently extracted data. Conflicts during the screening and extraction process were resolved by discussion and by involvement of a third reviewer.

The study was prospectively registered at PROSPERO as CRD42024523299), and the protocol was recently published [11].

### Data analysis and synthesis

Owing to the heterogeneity of the included studies—in terms of populations, interventions, comparisons, and outcomes—we did not perform a quantitative meta-analysis but provide results in summary tables and a qualitative narrative synthesis, grouping outcome criteria to address evidence gaps.

### Quality assessment

Quality assessment of all studies was carried out by 2 authors separately using the revised Grading of Recommendations, Assessment, Development, and Evaluation (GRADE) Cochrane risk of bias tool for randomized trials (RoB 2.0 tool) [13] and Critical Appraisal Skills Programme (CASP) [14] for observational studies. We created a risk of bias score for each study using standard terms and colors: low risk (green), some risk (yellow), and high risk (red). We analyzed the “Transparency of the Methodology and Results” for both randomized controlled trials (RCTs) and observational studies, categorizing them as yes (coded 2), no (coded 0), or unsure (coded 1). We then created a summary score, which we grouped into potential quartiles and around the categorical classifications of excellent, which refers to the scores that fell within the highest or fourth quartile; good, which refers to the scores in the third quartile; fair, which refers to the scores in the second quartile; and poor, which refers to the scores within the first quartile regarding transparency. Regarding the bias classification, we determined low bias (green) to be the categories of either excellent or good levels of transparency and high bias (red) to be the categories of either fair or poor exclusively. If there was a combination of excellent/good and fair/poor in the classifications for transparency and bias, we deemed some risk (yellow) as the final category.

CASP classification for observational studies was implemented by adding a supplementary classification of ROBINS-E–based analysis of confounding [15], which was published after the original submission of our PROSPERO protocol. The original tool already covered 6 of 7 ROBINS-E domains, including measurement of the exposure, selection of the participants, postexposure interventions, missing data, measurement of outcomes, and selection of the reported results. In order to maintain coherent grading for RCTs and observational studies, we recoded ROBINS-E grading on a 0–2 point scale, where 0 stands for “very high” and “high,” 1 stands for “some concerns,” and 2 stands for “low.”

The complete scores can be viewed in Supplementary Tables 2–7, with some categories not being applicable for some articles. Quartiles were determined from each article using these scores.

### GRADE for certainty of the evidence

The overall level of certainty (high, moderate, low, or very low) was computed using the Effective Practice and Organization of Care rules [16]: initial confidence was 4 points for RCTs and 2 points for nonrandomized studies; then, deductions were made of −2 points for a very serious flaw and −1 point for a serious flaw in all 5 dimensions (limitations of the design or execution of randomized trials, inconsistency, indirectness, imprecision, and dissemination bias) (see Supplemental Table 8).

## Results

**FIGURE 1:**
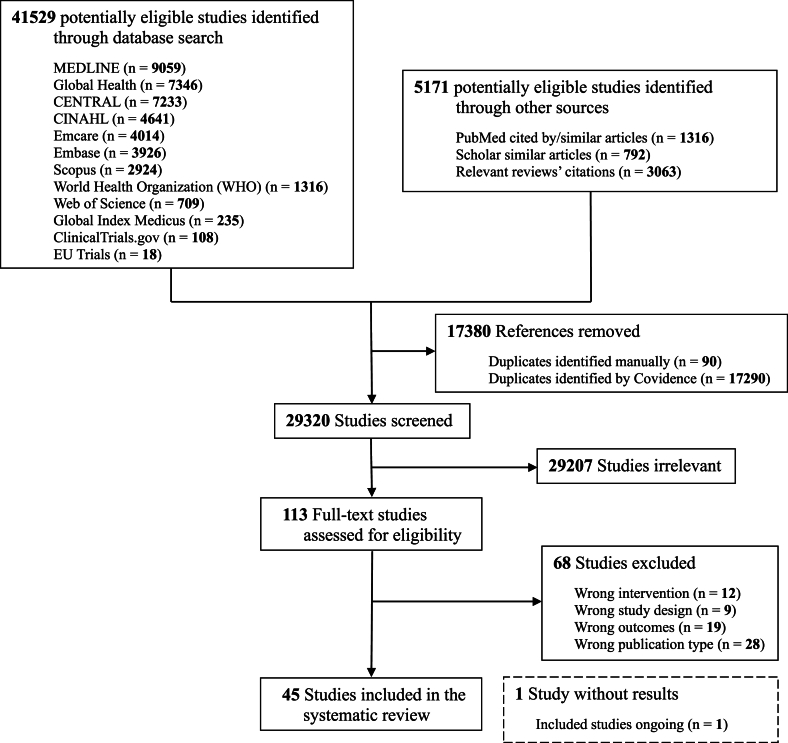
PRISMA flow diagram of retrieved literature on the influence of feeding expressed mother’s own milk using varying expression practices or treatments on health and growth of recipient infants.

We identified 46,700 records, resulting in 29,320 unique records for screening. Of these, 113 records were found to be relevant for full-text screening. Ultimately, 45 records were eligible for inclusion (Figure 1).

**FIGURE 2:**
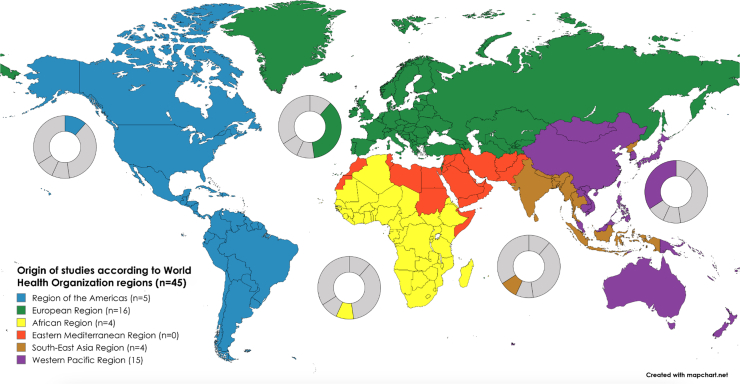
Studies included in the systematic review classified according to WHO regions.

Characteristics of the included studies are summarized in Table 2 [3,4,[17], [18], [19], [20], [21], [22], [23], [24], [25], [26], [27], [28], [29], [30], [31], [32], [33], [34], [35], [36], [37], [38], [39], [40], [41], [42], [43], [44], [45], [46], [47], [48], [49], [50], [51], [52], [53], [54], [55], [56], [57], [58], [59]]. Just under half of the studies (*n* = 21) were RCTs, and just over half of the studies (*n* = 24) were observational studies (18 prospective cohort studies, 4 retrospective cohort studies, and 2 prospective descriptive studies). Most studies were based in a single setting, and 10 studies had multiple settings, ranging from 2 to 25 settings. Most studies were conducted in the European Region and in the Western Pacific Region (Figure 2).

**TABLE 2:** Basic characteristics of the included studies.

Study	Study design	Setting (country, years)	Sample size (*n*)	Population (GA category/birth weight category)	Interventions	Comparison/controls	Outcomes
Intervention 1: MOM is expressed using different methods							
Alshaikh et al. [17]	Single-center prospective cohort study	Canada 2019–2022	34	VLBW	Hindmilk fraction of MOM (+ standard fortification)	Composite MOM (+ standard fortification)	Growth^1^; plasma fatty acids
Anderson et al. [18]	Single-center parallel RCT	Australia 2018–2019	57	Very preterm, moderately preterm	Breast massage + hand expression within 1 h of birth	Hand expression within 6 h of birth	Breastfeeding
Boo et al. [19]	Single-center parallel RCT	Malaysia 2000–2001	28	VLBW	Manual pump expression	Hand expression	Mortality; breastfeeding; length of hospital stay
Burton et al. [20]	Multicenter parallel RCT	United Kingdom	71	Extremely preterm, very preterm, moderately preterm	Electric pump expression—Philips Avent Twin electric pump	Electric pump expression—Medela Symphony pump	Breastfeeding
Fewtrell et al. [21]	Single-center parallel RCT	United Kingdom 1998–2000	166	Extremely preterm, very preterm, moderately preterm	Electric pump expression	Hand expression	Respiratory outcomes; NEC; other infections; breastfeeding
Fewtrell et al. [22]	Multicenter parallel RCT	United Kingdom, Russia, China, United States	112	Term	Electric pump expression—Philips single-electric pump, natural bottle	Electric pump expression—Medela Swing single-electric pump, Calma bottle	Breastfeeding
Flaherman et al. [23]	Multicenter parallel RCT	United States 2007–2009	68	Term	Electric pump expression	Hand expression	Breastfeeding
Fok et al. [24]	Single-center parallel RCT	Singapore	60	Term	Electric pump expression—scheduled expression regimen	Electric pump expression—free expression regimen	Growth; breastfeeding
Hayes et al. [25]	Multicenter parallel RCT	Hawaii, United States 2002–2003	246	Term	Electric pump expression	Manual pump expression	Breastfeeding^1^
Kalathingal et al. [26]	Single-center parallel RCT	India 2021–2022	78	VLBW	Electric pump expression—special expression regimen (“power pumping”)	Electric pump expression—regular expression regimen	Growth; breastfeeding^1^; length of hospital stay
Ogechi et al. [27]	Single-center parallel RCT	Nigeria 2000–2001	77	VLBW	Hindmilk fraction of MOM	Composite MOM	Growth^1^
Slusher et al. [28]	Single-center prospective cohort study	Nigeria 1999	16	Low birth weight	Hindmilk fraction of MOM	Composite MOM	Growth^1^
Zhou et al. [29]	Single-center parallel RCT	China 2018–2019	154	Late-preterm, term	Simultaneous pumping (Avent SCF303/01 bilateral electric breast pumps, Philips Invest, China)	*1*) Sequential pumping (Avent SCF902/11 single electric breast pump, Philips Invest, China); *2*) hand expression	Breastfeeding^1^
Intervention 3: MOM is treated with different methods							
Andersson et al. [30]	Single-center crossover RCT	Sweden	5	VLBW	HoP MOM	Raw MOM	Growth^1^
Ando et al. [31]	Single-center prospective descriptive study	Japan	44	—	Frozen-thawed MOM	—	HTLV-1 infection^1^
Ando et al. [32]	Single-center prospective cohort study	Japan 1985–1987	83	—	Frozen-thawed MOM	Fresh MOM	HTLV-1 infection^1^
Balcells et al. [33]	Multicenter prospective cohort study	Spain 2013–2014	1325	VLBW	Frozen-thawed MOM	Fresh MOM	CMV infection^1^
Bapistella et al. [3]	Multicenter (+1 historical control) prospective cohort study	Germany 2010–2012	196	VLBW	Short-term pasteurized MOM (62°C for 5 s)	Raw MOM	NEC; focal intestinal perforation; IVH/PVL; ROP; CMV infection
Bedwell et al. [34]	Single-center parallel RCT	United States	44	Extremely preterm, very preterm	Continuous warming (Medela Guardian milk warmer)	Standard warming (hot water bath)	Growth^1^
Chantry et al. [35]	—	Tanzania 2008–2009	144	—	Flash-heated MOM	Raw MOM	Growth; other infections; breastfeeding
Chiavarini et al. [36]	Single-center prospective descriptive study	Italy 2004–2007	57	Extremely preterm, very preterm, low birth weight	Frozen-thawed MOM	—	CMV infection^1^
Chung et al. [4]	Multicenter parallel RCT	South Korea 2015–2019	125	Extremely preterm, very preterm, VLBW	*1*) Frozen-thawed and HTST pasteurized MOM (72 °C for 5 s); *2*) Frozen-thawed and low-temperature HoP MOM	Frozen-thawed MOM	Mortality; respiratory outcomes; NEC; ROP; feeding tolerance; CMV infection^1^; other infections
Cossey et al. [37]	Single-center parallel RCT	Belgium 2006–2010	303	Extremely preterm, very preterm, VLBW	HoP MOM	Raw MOM	Growth; mortality; respiratory outcomes; NEC; IVH/PVL; ROP; feeding tolerance; sepsis^1^; length of hospitalization
de Halleux et al. [38]	Single-center prospective cohort study	Belgium 2007–2014	101	Extremely preterm, very preterm	HoP MOM	Raw MOM	Growth^1^
de Oliveira et al. [39]	Single-center crossover RCT	France 2014–2015	14	Very preterm	HoP MOM	Raw MOM	Gastric digestion^1^
Dicky et al. [40]	Multicenter prospective cohort study	France 2011	926	Extremely preterm, very preterm	HoP MOM	Raw MOM	Growth^1^; mortality^1^; respiratory outcomes^1^; NEC^1^; feeding tolerance^1^; sepsis^1^; length of hospital stay^1^
Gang and Chang [41]	Single-center retrospective descriptive study	South Korea 2013–2017	232	Extremely preterm, very preterm, VLBW	Frozen-thawed and HoP MOM	—	CMV infection^1^
Hayashi et al. [42]	Multicenter prospective descriptive study	Japan 2003–2004	27	Extremely preterm, extremely low birth weight	Frozen-thawed MOM	—	CMV infection^1^
Huang et al. [43]	Single-center prospective cohort study	China 2018–2020	171	Extremely preterm, very preterm, VLBW	HoP MOM	Fresh MOM	Growth; mortality; respiratory outcomes; NEC; IVH/PVL; ROP; feeding tolerance; adverse events; other infections; survival without severe complications
Hung et al. [44]	Single-center crossover RCT	Taiwan 2009	18	Extremely preterm, very preterm, moderately preterm	Frozen-thawed MOM	Fresh MOM	Stress behaviors
Jim et al. [45]	Single-center prospective descriptive study	Taiwan 2000-2002	42	VLBW	Frozen-thawed MOM	—	CMV infection^1^
Joachim [46] (ongoing)	Single-center prospective cohort study	Germany 2021–2023	29	Extremely low birth weight	Pasteurized MOM	Raw MOM	Growth; feeding tolerance; microbiota composition
Mbuya et al. [47]	Single-center prospective descriptive study	Zimbabwe 2008–2009	20	—	Flash-heated MOM	—	Growth
Merter and Altay [48]	Single-center prospective cohort study	Turkey 2018–2020	40	Very preterm	Frozen-thawed MOM	Fresh MOM	Microbiota composition^1^
Narayanan et al. [49]	Parallel RCT (number of centers not reported)	India	226	Low birth weight	*1*) Raw MOM; *2*) pasteurized MOM (62.5°C for 40 min)	*1*) Raw MOM + formula; *2*) pasteurized MOM + formula	Sepsis^1^; length of hospital stay
Ogawa et al. [50]	Single-center prospective descriptive study	Japan 2017–2021	136	Extremely preterm, very preterm	Frozen-thawed MOM	—	CMV infection^1^
Omarsdottir et al. [51]	Multicenter parallel RCT	Sweden 2005–2009	140	Extremely preterm	Frozen-thawed MOM	Fresh MOM + frozen-thawed MOM	Growth; mortality; respiratory outcomes; CMV infection^1^; other infections; feeding tolerance; breastfeeding; cardiovascular outcomes; cholestasis; blood transfusions; plasma transfusions; length of hospital stay
Stock et al. [52]	Single-center retrospective cohort study	Austria 2008–2013	341	Extremely preterm, very preterm	HoP MOM	Raw MOM	Mortality; NEC^1^; IVH/PVL; feeding tolerance; CMV infection; other infections^1^; blood transfusions; length of hospital stay
Sun et al. [53]	Multicenter prospective cohort study	China 2016–2017	221	Extremely preterm, very preterm	Frozen-thawed MOM	Fresh MOM	Growth^1^; mortality, respiratory outcomes; NEC; IVH/PVL, ROP; feeding tolerance; other infections
Thomaz et al. [54]	Single-center crossover RCT	Brazil	45	Very preterm, moderately preterm, VLBW	*1*) Pasteurized homogenized MOM; *2*) pasteurized nonhomogenized MOM	*1*) Raw homogenized MOM; *2*) raw nonhomogenized MOM	Fat absorption
Uygur et al. [55]	Single-center parallel RCT	Turkey 2012–2014	80	VLBW	Fresh MOM at 32–34°C	Fresh MOM at 22–24°C	Growth; NEC; feeding tolerance; gastroesophageal reflux; apneas
Volder et al. [56]	Single-center prospective descriptive study	Denmark 2019–2020	26	Extremely preterm, very preterm	Fresh MOM for first 14 d of life, then combination of frozen-thawed MOM and fresh MOM	—	CMV infection^1^
Wakabayashi et al. [57]	Single-center prospective descriptive study	Japan 2010–2011	11	VLBW	Frozen-thawed MOM	—	Growth; CMV infection
Yoo et al. [58]	Single-center retrospective cohort study	South Korea 2007–2013	385	Extremely low birth weight	HoP MOM	Frozen-thawed MOM	Growth; mortality; respiratory outcomes; NEC; IVH/PVL; feeding tolerance; CMV infection^1^; other infections; length of hospital stay
You et al. [59]	Single-center retrospective cohort study	China 2018–2020	103	Extremely preterm, very preterm, VLBW	Frozen-thawed MOM	Fresh MOM	Respiratory outcomes; NEC; ROP; feeding tolerance; CMV infection^1^; other infections

Abbreviations: CMV, cytomegalovirus; GA, gestational age; HoP, holder-pasteurized; HTST, high-temperature short-time; HTLV, human T-cell leukemia/lymphoma virus; IVH, intraventricular hemorrhage; MOM, mother’s own milk; NEC, necrotizing enterocolitis; PVL, periventricular leukomalacia; RCT, randomized controlled trial; ROP, retinopathy of prematurity; VLBW, very low birth weight.

1 Primary outcome.

Thirteen studies investigated methods of milk expression, and 32 studies investigated treatments of MOM. No study evaluated hygiene practices or settings used during milk expression. The evidence gap map is available in Supplemental Figure 1.

The risk of bias analysis was conducted on all included studies (21 RCTs and 24 observational studies) and is included in Supplemental Tables 2 and 5. Most biases were due to methodological issues, such as small sample sizes. We rated 16 RCTs and 14 observational studies as having low risk of bias.

### Intervention 1: MOM is expressed using different methods

Four RCTs focused on different expression regimens [18,24,26,29]. Evaluated outcomes included breastfeeding [18,26,29], infant growth [24,26], and length of hospitalization [26]. In all 4 studies, the evaluated expression regimens did not result in significant differences of the aforementioned outcomes.

Three RCTs compared pump expression with hand expression in the early postpartum period [19,23,29]. Flaherman et al. [23] found a higher breastfeeding rate at 2 months in mothers of term infants assigned to hand expression, whereas Zhou et al. [29] did not detect any significant difference in breastfeeding rates. No difference in NEC or sepsis was detected [19], although the high mortality rate, unusual frequency of bacteremia and NEC, and unacceptable level of contamination of all samples (either collected at home or in the hospital) required the enrolled infants to be fed with pasteurized milk from month 4.

Four RCTs compared different models of pumps. Two studies compared the use of manual pumps and electric pumps and found no significant difference in breastfeeding status at 6 mo [21,26], need for supplemental oxygen or ventilation, NEC, and number of courses or total number of days of antibiotics received [21]. Two studies compared different electric pumps; no difference in breastfeeding rate was detected in term infants [22], whereas a significant improvement was seen in the likelihood of direct breastfeeding at discharge for preterm infants using the novel pump equipped with petal cushions [20].

Three studies compared the effect of feeding hindmilk with composite milk on growth of preterm infants [17,27,28]. In the study by Alshaikh et al. [17], both intervention groups received standard milk fortification as well. Hindmilk was associated with improved weight gain [17,27,28] but infant length [17,27] and was associated with higher plasma levels of linoleic acid and α-linoleic acid [17] without affecting metabolic acidosis [27].

Although both outcomes described for hindmilk (growth and nutrient deficiencies) were graded as low certainty (Supplemental Table 8), due to the effects of serious imprecision, indirectness, and inconsistency, all the outcomes related to pumping method or regimen, namely, breastfeeding rates (evaluated by 8 RCTs), growth (2 RCTs), and morbidity (1 RCT), were graded as of high certainty.

### Intervention 2: MOM is expressed with different settings and/or hygiene practices

We did not find any study evaluating the effect on recipient infants of different hygiene practices or settings during MOM expression.

### Intervention 3: MOM is treated with different methods

Thirty-two studies investigated different processing methods of MOM, including freeze-thawing (*n* = 14), heat treatments (*n* = 17), and warming methods (*n* = 2).

Among studies evaluating freeze-thawing, 5 studies compared frozen-thawed MOM with fresh MOM [32,33,44,48,59] and 2 studies with a combination of fresh MOM and frozen-thawed MOM [51,53].

Sun et al. [53] found that infants fed >1 daily serving of fresh MOM gained weight faster than infants always fed frozen milk, whereas Omarsdottir et al. [51] found no difference in weight at 36 wk. In the study by Wakabayashi et al. [57], CMV-infected infants fed >1 daily serving of fresh MOM and symptomatic for CMV infection were found to have significantly slower growth than nonsymptomatic infants on the same diet.

Mortality was found to be similar between infants fed only frozen-thawed and those receiving ≥1 daily serving of fresh MOM [51,53].

Morbidity was reported as possibly influenced by the use of ≥1 daily serving of fresh MOM. Although IVH (grade ≥3) was not influenced, a lower composite risk for NEC stage ≥3 or mortality, sepsis, ROP, and BPD was reported [53]. Another study reported no difference in the same outcomes, except a significant increase in fungal sepsis with fresh MOM [51]. No difference was found for NEC, BPD, ROP, and sepsis in a third study investigating exclusive fresh MOM diet compared with exclusive frozen MOM diet [59]. No difference was reported for patent ductus arteriosus requiring treatment, inotropic support, neonatal cholestasis, blood or plasma transfusions, or length of hospitalization [51].

Preterm infants showed more stress cues during feeding when receiving frozen-thawed MOM (−13°C for 5 d) compared with fresh MOM [44] and a different intestinal microbiota composition [48]: there were more *Lactobacillus* and Bifidobacteria in the fresh MOM group and more Streptococci and Enterobacteriaceae in the freeze-thawed MOM group.

Feeding tolerance was found to be similar by Omarsdottir et al. [51] and You et al. [59]. However, Sun et al. [53] found that infants fed fresh MOM received parenteral nutrition for a shorter time.

When freeze-thawing was evaluated for viral infection prevention, a 12-h cycle was successful in reducing viral transmission to infants from HTLV-1–infected mothers [31,32]. For CMV infection, freezing was usually performed for >72 h. Balcells et al. [33] found that feeding freeze-thawing MOM resulted in a 78% reduction of acquired CMV when compared with fresh MOM [relative risk: 0.22; 95% confidence interval (CI): 0.05, 0.90]. Conversely, Omarsdottir et al. [51] found no difference in CMV transmission rate in extremely preterm infants. Rate of CMV transmission in infants fed frozen-thawed MOM in included studies is shown in Table 3 [3,4,33,36,41,42,45,[50], [51], [52],[56], [57], [58], [59]]. Higher values were reported by 2 studies that provided infants with a mixed fresh/frozen-thawed diet [56,57].

**TABLE 3:** Postnatal CMV transmission for intervention 3: freeze-thawing and/or heat treatment.

Study	Country	Maternal CMV status	Sample size (*n*)	Infant GA (wk), mean ± SD (range)	Infant BW (g), mean ± SD (range)	MOM treatment	Rate of postnatal infection (%)
Balcells et al. [33]	Spain	Any	166	29.5 ± 2.4	1125 ± 251	Freeze-thawing	2
Bapistella et al. [3]	Germany	IgG+	87	29.7	1130	HTST	2.3
Chiavarini et al. [36]	Italy	IgG+	57	29 (23–34)	1158 ± 436.3	Freeze-thawing	2.5
Chung et al. [4]	South Korea	CMV+ MOM	41	28.3 ± 2.9	1090.9 ± 399.3	Freeze-thawing	4.9
			42	27.7 ± 2.9	1017.9 ± 368.3	HoP	9.5
			42	27.8 ± 2.5	1020.8 ± 288.0	HTST	2.4
Gang and Chang [41]	South Korea	Any	63	NR	NR	Freeze-thawing + HoP	6.3
Hayashi et al. [42]	Japan	IgG+	27	26.4 (23.7–32.6)	802 (512–1108)	Freeze-thawing	4.3
Jim et al. [45]	Taiwan	IgG+	40	Not infected: 29.6 ± 2.3 Infected: 30.7 ± 1.5	Not infected: 1200 ± 200 Infected: 1300 ± 200	Freeze-thawing	15
Ogawa et al. [50]	Japan	Any	139	Not infected: 28.3 Infected: 25.5	Not infected: 993 Infected: 802	Freeze-thawing	5
Omarsdottir et al. [51]	Sweden	Any	53	25.9 ± 1.2	846 ± 178	Freeze-thawing	5.7
Stock et al. [52]	Austria	Any	53	25.9 ± 1.2	846 ± 178	Freeze-thawing	5.7
Volder et al. [56]	Denmark	IgG+	16	28.1	1074	Freeze-thawing	25
Wakabayashi et al. [57]	Japan	Any	11	27 (24–33)	864 (615–1418)	Freeze-thawing	45.5
Yoo et al. [58]	South Korea	Any	323	NR	NR	Freeze-thawing	8
			62	25.4 ± 1.1 (23.3–30.1)	685 ± 166 (320–990)	HoP	0
You et al. [59]	China	CMV+ MOM	67	Not infected: 30.0 (27.2–36.2) Infected: 28.5 (28.0–29.3)	Not infected: 1260 (720–1700) Infected 1200 (1150–1610)	Freeze-thawing	9

Abbreviations: BW, birth weight; CMV, cytomegalovirus; GA, gestational age; HoP, holder pasteurization; HTST, high-temperature short-time pasteurization; MOM, mother’s own milk; NR, not reported.

Seven of the outcomes described for freeze-thawing of MOM are of low or moderate certainty (Supplemental Table 8). Three studies (1 RCT and 2 observational) on morbidity revealed serious imprecision, but other domains evaluated had positive findings, leading to an overall moderate certainty score. One RCT and 1 observational study on mortality were affected only by imprecision and were graded as having moderate certainty. CMV infection studies included 1 RCT and 7 observational studies with a high level of inconsistency and imprecision; therefore, the overall score was low. For the other infections, we found 1 RCT and 2 observational studies, also affected by serious inconsistency and imprecision of the results; therefore, we graded this outcome as having low certainty. In 2 observational studies measuring retroviral infection (HTLV-1), only imprecision was problematic, but because of the research design, we deemed retroviral infection to have low certainty. The results from the 3 studies evaluating growth (1 RCT and 2 observational) were affected by the high levels of inconsistency and imprecision and thus graded as having low certainty. Feeding tolerance, which was described in 1 RCT and 2 observational studies, was affected by a serious level of inconsistency and imprecision; therefore, the final grade was low.

Eleven studies evaluated holder-pasteurized (HoP) MOM and 2 studies evaluated high-temperature short-time (HTST) pasteurized MOM.

Growth was not affected by pasteurization in 4 studies [30,37,40,58]. De Halleux et al. [38] found that weight gain, but not weight *z*-score gain, was significantly higher in the raw MOM group than in the HoP MOM group. Length and head circumference (HC) gains were similar. Huang et al. [43] found that the fresh MOM group had a shorter time to regain birth weight compared with the HoP MOM group but that both groups had same weight, length, and HC velocity.

Mortality was not found to be affected by pasteurization of MOM [4,37,40,43,52,58]. However, Huang et al. [43] found that infants fed fresh MOM had a higher survival rate without severe complications than that of the HoP MOM group.

Some studies reported higher BPD incidence in infants receiving HoP MOM than in those receiving fresh MOM [40,43], whereas others found no significant differences among HoP, frozen-thawed, HTST pasteurized, or raw MOM [4,37]. Yoo et al. [58] noted longer oxygen therapy with frozen-thawed MOM than with HoP MOM.

NEC was not found to be affected by pasteurization [3,4,37,40,43,52,58]. ROP was not found to be affected by pasteurization [4,37,43,58], except in 1 study [3], which found a lower ROP rate in infants fed HTST pasteurized MOM than in the historical cohort fed with raw MOM. IVH/PVL was not found to be affected by pasteurization [3,37,43,52,58].

Most studies found similar feeding tolerance across pasteurized, raw, and frozen-thawed MOM, although 2 reported faster progression with fresh/raw MOM than with pasteurized MOM [43,52].

de Oliveira et al. [39] found that HoP MOM did not affect gastric emptying or pH. The only study reporting adverse events observed none in either group [43].

Pasteurization was effective in reducing the risk of postnatal CMV infection. Bapistella et al. [3] reported a risk ratio for infants fed raw to HTST pasteurized MOM of 8.3 (95% CI: 2.4, 52.4). HoP MOM was more effective than feeding raw [52] or freeze-thawed MOM [58] in reducing postnatal CMV infection. In contrast, Chung et al. [4] reported similar rates of CMV transmission maong infants fed frozen-thawed, HoP, and HTST pasteurized MOM.

Incidence of low-onset sepsis was not found to be affected by pasteurization [4,37,40,43,49,52,58]. Length of hospitalization was not affected by pasteurization either [37,40,49,52].

Flash-heated MOM was associated with similar growth to raw MOM in HIV-exposed infants, with no growth faltering observed [35,47] and no effect on infection rates [35].

Two studies evaluated methods of milk warming [34,55]. Bedwell et al. [34] found higher weight gain with continuous warming than with standard water-bath warming. Uygur et al. [55] found no differences in time to regain birth weight, daily weight gain, NEC, or time to full enteral feeding across different milk temperatures, although apnea and antireflux treatment were more frequent at 22–24°C than at 32–34°C.

Ultrasonic homogenized MOM, whether raw or pasteurized, was associated with higher fat absorption compared with nonhomogenized milk [54].

The heat treatment intervention was measured by 7 outcomes. Growth was studied in 2 RCTs and 7 observational studies. Although not affected by any serious flaw, because of the high number of observational studies included, this outcome was graded as having moderate certainty (Supplemental Table 8). The same observation on the balance between observational studies and RCTs affected the grading of other outcomes, such as mortality, morbidity, CMV infection, and feeding tolerance. Adverse events were measured in 1 observational study, which was affected by serious imprecision and was thus classified as having very low certainty.

## Discussion

This systematic review comprehensively evaluated the effects of different expression practices and processing techniques for MOM on a broad range of clinical outcomes in recipient infants. Unlike previous reports, which have largely focused on compositional changes, this work examined outcomes of direct clinical relevance (growth, major morbidities, mortality, feeding tolerance, infections, adverse events, and breastfeeding). Moreover, by considering expressed MOM and excluding donor milk or direct breastfeeding, this review addresses a distinct gap in the literature with important implications for neonatal care.

Regarding methods of expression, no particular expression regimen or pump type proved superior for infant growth or breastfeeding outcomes. Hand expression was at least as effective as electric or manual pumping and, in 1 study, was associated with improved breastfeeding continuation at 2 mo. These findings support hand expression as an efficient alternative to pumping, especially in low-resource settings.

The evidence on hindmilk was more consistent, although graded as having low certainty. Three studies demonstrated improved weight gain in preterm infants fed mother’s hindmilk rather than composite MOM. This aligns with known differences in hindmilk composition—higher fat content [60]—and suggests that hindmilk use may represent an effective additional feeding strategy for infants with suboptimal growth, both in low- and high-resource settings.

For processing methods, results were heterogeneous. Freeze-thawing did not appear to affect mortality or major morbidities; however, most studies were underpowered to evaluate those outcomes. Effects on growth and feeding tolerance were inconsistent. Limited direct evidence was found for the efficacy of freeze-thawing in reducing CMV transmission to infants, with inconclusive results. Nevertheless, lower CMV viral loads are well documented after freeze-thawing, consistent with the meta-analysis by Park et al. [61], which reported higher CMV infection rates in preterm infants fed fresh milk than in those fed frozen-thawed milk. Overall, the use of mixed fresh/frozen MOM diets seems to affect the CMV transmission rates.

Our review found that pasteurization, particularly HoP MOM, consistently reduced CMV transmission compared with raw or frozen-thawed MOM but showed no clear effect on mortality, NEC, sepsis, or other morbidities. Again, most studies were underpowered for these outcomes. Two studies suggested reduced feeding tolerance with pasteurized compared with fresh/raw MOM. This observation supports the superiority of feeding fresh milk over thermally processed milk whenever possible [62]. The systematic review by Gomez et al. [63] based on 9 studies also suggested a better growth rate and feeding tolerance as well as a lower rate of BPD in infants fed raw MOM than in those fed processed MOM. Human milk processing was not associated with an increased risk of ROP in our review. Evidence from the meta-analysis by Bharvani et al. [64] further supports that providing any quantity of human milk, even if not exclusively, is clinically important for reducing the incidence of both any-stage ROP and severe ROP.

We also found limited evidence on the influence of milk processing on infant growth, which, as far as we know, depends not only on the concentration of specific nutrients in the processed milk but also on their quality and bioavailability [9,65]. Evidence on the growth and health outcomes for alternative processing methods (high-pressure processing, UV-C, retort pasteurization) is not available to date because these methods are not intended at present for MOM but for use in donor human milk banks.

Overall, the certainty of evidence from the retrieved literature was low. Most studies were small and heterogeneous in design, population, interventions, and outcomes. Many outcomes were reported inconsistently, and, notably, for mortality and major morbidities such as NEC, most studies were underpowered to detect realistic differences. Therefore, reported findings of “no difference” must be interpreted with caution because they might possibly reflect an insufficient sample size rather than equivalence between interventions. Besides, important areas of intervention have not been targeted by research to date, such as the effects of different hygiene practices during milk expression. Moreover, neurodevelopmental outcomes were not reported in any retrieved article. Addressing these gaps in future studies will be essential to optimize infant health outcomes.

In summary, although some practices— notably, hindmilk feeding for infant growth and pasteurization for CMV prevention—were associated with consistent or promising benefits, the overall evidence remains limited. The reviewed studies shared several methodological weaknesses: most relied on convenience or purposive sampling, few reported sample size calculations or tested group comparability, and clinical characteristics were often incompletely described. Small sample sizes limited statistical power and frequently resulted in basic analyses without effect sizes or CIs, reducing the reliability and generalizability of findings.

The small number of eligible studies, combined with marked heterogeneity in populations, interventions, and outcomes, also precluded quantitative synthesis. Evidence from low-resource settings was particularly scarce. We tried to minimize the selection bias through a broad search strategy that included non-English studies and gray literature; however, some studies—particularly those published in local journals without English abstracts—may have been missed.

Future research should address these limitations through adequately powered, multicenter studies with robust methodology and standardized outcome reporting. Priorities include morbidity, mortality, and long-term neurodevelopmental outcomes, as well as hygiene practices and storage conditions during milk expression, which remain unstudied. Addressing these gaps will be essential to provide more reliable evidence and guide best practice in neonatal care.

## Author contributions

The authors’ responsibilities were as follows – AW, TC, SG, DK: originally conceptualized the present manuscript; CS, BW: provided software expertise; TC, BW, MG, LC, AW: validated the data; TC, BW: performed formal analysis; AW: was in charge of resources, project administration, and supervision; MG, CS, TC: curated the data; TC, SG, MG: performed visualization of the data; AW, SG, AB-J, TC, DK: acquired funding; and all authors: read and approved the final manuscript.

## Data availability

Data described in the article, code book, and analytic code will be made available upon request.

## Funding

The present systematic review was supported by the WHO Department of Nutrition and Food Safety, as a response to the “Call for authors—Systematic reviews on donor human milk banking processes” (deadline of submission: 5 November, 2023). The sponsors provided guidance and supervision in the study design, data collection, data analysis, data interpretation, and writing of the report.

## Conflict of interest

All authors report financial support from WHO. MG and LC report consulting or advisory roles for Labor Baby srl and have patent #EP 2974 603 B1 licensed to Labor Baby srl. AW is a coinvestigator of the Polish patent (nr Pat.238537, submission number P.429126) concerning the optimization of high-pressure preservation of human milk, within the framework of the uncommercial research financed “Lactotechnology as a response for vulnerable baby” by the National Centre for Research and Development for non-governmental organizations.
