# Supplementary material for: A Systematic Review on the Influence of Feeding Expressed Mother’s Own Milk Using Varying Expression Practices or Treatments on Health and Growth of Recipient Infants
Source: Adv Nutr. 2025 Sep 19;16(11):100523. doi: 10.1016/j.advnut.2025.100523 (PMC12550144; doi:10.1016/j.advnut.2025.100523)
Supplement: Multimedia component 1 [file mmc1.docx]

**Supplemental Table 1: Detailed search strings and results**

|  | **Search Strategy for CINAHL** | **Results** |
| --- | --- | --- |
| S1 | (MM "Milk, Human") OR (MM "Breast Feeding") OR (MM Lactation+) | 22.627 |
| S2 | (express* OR pump* OR extract* OR collect* OR treat* OR pasteuri* OR heat OR thermal OR composition OR process* OR microbial OR antimicrobial OR contaminat* OR freez* OR froze* OR sonic* OR UV OR hygien* OR save* OR saving OR stor* OR keep* OR conserv* OR preserv* OR Unpasteuri* OR Steril* OR microbiolog* OR bacteria* OR cool* OR macronutrient* ) | 2.747.795 |
| S3 | S1 AND S2 | 7.905 |
| S4 | ((breastmilk OR ((breast OR human OR mother* OR parent OR parents ) AND milk ) OR breastfe* OR "breast feed*" OR "breast fed" OR colostrum OR lactat* ) N3 (express* OR pump* OR extract* OR collect* OR treat* OR pasteuri* OR heat OR thermal OR composition OR process* OR microbial OR antimicrobial OR contaminat* OR freez* OR froze* OR sonic* OR UV OR hygien* OR save* OR saving OR stor* OR keep* OR conserv* OR preserv* OR Unpasteuri* OR Steril* OR microbiolog* OR bacteria* OR cool* OR macronutrient* )) | 6.568 |
| S5 | (MH "Milk Expression") | 749 |
| S6 | "hand express*" | 70 |
| S7 | S3 OR S4 OR S5 OR S6 | 10.834 |
| S8 | (MM Infant+) | 23.549 |
| S9 | (Outcome* OR impact* OR affect* OR influenc* OR result* OR consequenc* OR caus* OR issue* OR threat* OR effect* OR benefit* OR harm* OR "risk factor*" OR tolera* OR growth* OR development* OR neurodevelop* OR behavio* OR weight OR digest* OR diet OR nutrition OR malnutrition OR deficien* OR illness* OR death* OR morbidity OR mortality OR infection* OR transmi* OR sepsis* OR enterocolitis* OR cytomegalovirus* OR HIV OR immunologic OR diarrh* OR meningitis OR "urinary tract infection*" OR UTI OR Expos* OR Quality OR Complication* ) | 5.397.922 |
| S10 | S8 AND S9 | 21.164 |
| S11 | ((infant* OR baby OR babies OR neonate* OR "new born*" OR newborn* OR preterm OR pre-term OR child* ) N4 (Outcome* OR impact* OR affect* OR influenc* OR result* OR consequenc* OR caus* OR issue* OR threat* OR effect* OR benefit* OR harm* OR "risk factor*" OR tolera* OR growth* OR development* OR neurodevelop* OR behavio* OR weight OR digest* OR diet OR nutrition OR malnutrition OR deficien* OR illness* OR death* OR morbidity OR mortality OR infection* OR transmi* OR sepsis* OR enterocolitis* OR cytomegalovirus* OR HIV OR immunologic OR diarrh* OR meningitis OR "urinary tract infection*" OR UTI OR Expos* OR Quality OR Complication* )) | 359.148 |
| S12 | ((infant* OR baby OR babies OR neonate* OR "new born*" OR newborn* OR preterm OR pre-term OR child*) OR (MH Infant+)) AND (MH "Growth+") OR (MH "Infant Physiology") | 57.107 |
| S13 | S10 OR S11 OR S12 | 389.543 |
| S14 | S7 AND S13 | 4.719 |
| S15 | S14 NOT ((MH Animals) NOT ((MH Animals) AND (MH Humans))) | 4.642 |

|  | **Search Strategy for ClinicalTrials.gov** | **Results** |
| --- | --- | --- |
| ● | ("breastmilk" OR (("breast" OR "human" OR "mother*" OR "parent" OR "parents") AND "milk") OR "breastfe*" OR "breast feed*" OR "breast fed" OR "colostrum" OR "lactat*") AND ("express*" OR "pump*" OR "extract*" OR "collect*" OR "treat*" OR "pasteuri*" OR "heat" OR "thermal" OR "composition" OR "process*" OR "microbial" OR "antimicrobial" OR "contaminat*" OR "freez*" OR "froze*" OR "sonic*" OR "UV" OR "hygien*" OR "save*" OR "saving" OR "stor*" OR "keep*" OR "conserv*" OR "preserv*" OR "Unpasteuri*" OR "Steril*" OR "microbiolog*" OR "bacteria*" OR "cool*" OR "macronutrient*") AND ("Outcome*" OR "impact*" OR "affect*" OR "influenc*" OR "result*" OR "consequenc*" OR "caus*" OR "issue*" OR "threat*" OR "effect*" OR "benefit*" OR "harm*" OR "risk factor*" OR "tolera*" OR "growth*" OR "development*" OR "neurodevelop*" OR "behavio*" OR "weight" OR "digest*" OR "diet" OR "nutrition" OR "malnutrition" OR "deficien*" OR "illness*" OR "death*" OR "morbidity" OR "mortality" OR "infection*" OR "transmi*" OR "sepsis*" OR "enterocolitis*" OR "cytomegalovirus*" OR "HIV" OR "immunologic" OR "diarrh*" OR "meningitis" OR "urinary tract infection*" OR "UTI" OR "Expos*" OR "Quality" OR "Complication*") AND ("infant*" OR "baby" OR "babies" OR "neonate*" OR "new born*" OR "newborn*" OR "preterm" OR "pre-term" OR "child*") | 108 |

|  | **Search Strategy for Cochrane trials** | **Results** |
| --- | --- | --- |
| #1 | [mh ^"Milk, Human"] OR [mh ^"Breast Feeding"] OR [mh Lactation] | 4.293 |
| #2 | (express* OR pump* OR extract* OR collect* OR treat* OR pasteuri* OR heat OR thermal OR composition OR process* OR microbial OR antimicrobial OR contaminat* OR freez* OR froze* OR sonic* OR UV OR hygien* OR save* OR saving OR stor* OR keep* OR conserv* OR preserv* OR Unpasteuri* OR Steril* OR microbiolog* OR bacteria* OR cool* OR macronutrient* ) | 1.314.592 |
| #3 | #1 AND #2 | 2.464 |
| #4 | ((breastmilk OR ((breast OR human OR mother* OR parent OR parents ) AND milk ) OR breastfe* OR ("breast" NEXT feed*) OR "breast fed" OR colostrum OR lactat* ) NEAR/3 (express* OR pump* OR extract* OR collect* OR treat* OR pasteuri* OR heat OR thermal OR composition OR process* OR microbial OR antimicrobial OR contaminat* OR freez* OR froze* OR sonic* OR UV OR hygien* OR save* OR saving OR stor* OR keep* OR conserv* OR preserv* OR Unpasteuri* OR Steril* OR microbiolog* OR bacteria* OR cool* OR macronutrient* )) | 51.242 |
| #5 | [mh ^"Breast Milk Expression"] | 43 |
| #6 | ("hand" NEXT express*) | 39 |
| #7 | #3 OR #4 OR #5 OR #6 | 52.717 |
| #8 | [mh Infant] | 45.884 |
| #9 | (Outcome* OR impact* OR affect* OR influenc* OR result* OR consequenc* OR caus* OR issue* OR threat* OR effect* OR benefit* OR harm* OR ("risk" NEXT factor*) OR tolera* OR growth* OR development* OR neurodevelop* OR behavio* OR weight OR digest* OR diet OR nutrition OR malnutrition OR deficien* OR illness* OR death* OR morbidity OR mortality OR infection* OR transmi* OR sepsis* OR enterocolitis* OR cytomegalovirus* OR HIV OR immunologic OR diarrh* OR meningitis OR ("urinary tract" NEXT infection*) OR UTI OR Expos* OR Quality OR Complication* ) | 2.133.708 |
| #10 | #8 AND #9 | 45.883 |
| #11 | ((infant* OR baby OR babies OR neonate* OR ("new" NEXT born*) OR newborn* OR preterm OR pre-term OR child* ) NEAR/4 (Outcome* OR impact* OR affect* OR influenc* OR result* OR consequenc* OR caus* OR issue* OR threat* OR effect* OR benefit* OR harm* OR ("risk" NEXT factor*) OR tolera* OR growth* OR development* OR neurodevelop* OR behavio* OR weight OR digest* OR diet OR nutrition OR malnutrition OR deficien* OR illness* OR death* OR morbidity OR mortality OR infection* OR transmi* OR sepsis* OR enterocolitis* OR cytomegalovirus* OR HIV OR immunologic OR diarrh* OR meningitis OR ("urinary tract" NEXT infection*) OR UTI OR Expos* OR Quality OR Complication* )) | 109.811 |
| #12 | ((infant* OR baby OR babies OR neonate* OR ("new" NEXT born*) OR newborn* OR preterm OR pre-term OR child* ) OR [mh Infant]) AND [mh "Growth and Development"] | 12.714 |
| #13 | #10 OR #11 OR #12 | 130.764 |
| #14 | #7 AND #13 | 7.776 |
| #15 | #14 NOT ([mh ^Animals] NOT ([mh ^Animals] AND [mh ^Humans])) in Trials | 7.233 |

|  | **Search Strategy for EMBASE** | **Results** |
| --- | --- | --- |
| 1 | *breast milk/ or exp *Breast Feeding/ or exp *Lactation/ | 63.640 |
| 2 | (express* or pump* or extract* or collect* or treat* or pasteuri* or heat or thermal or composition or process* or microbial or antimicrobial or contaminat* or freez* or froze* or sonic* or UV or hygien* or save* or saving or stor* or keep* or conserv* or preserv* or Unpasteuri* or Steril* or microbiolog* or bacteria* or cool* or macronutrient*).ti,ab,kf. | 19.153.893 |
| 3 | 1 and 2 | 27.909 |
| 4 | ((breastmilk or ((breast or human or mother* or parent or parents) and milk) or breastfe* or breast feed* or breast fed or colostrum or lactat*) adj3 (express* or pump* or extract* or collect* or treat* or pasteuri* or heat or thermal or composition or process* or microbial or antimicrobial or contaminat* or freez* or froze* or sonic* or UV or hygien* or save* or saving or stor* or keep* or conserv* or preserv* or Unpasteuri* or Steril* or microbiolog* or bacteria* or cool* or macronutrient*)).ti,ab,kf. | 29.567 |
| 5 | Breast Milk Expression/ | 545 |
| 6 | hand express*.ti,ab,kf. | 658 |
| 7 | 3 or 4 or 5 or 6 | 48.036 |
| 8 | exp *Infant/ | 47.332 |
| 9 | (Outcome* or impact* or affect* or influenc* or result* or consequenc* or caus* or issue* or threat* or effect* or benefit* or harm* or risk factor* or tolera* or growth* or development* or neurodevelop* or behavio* or weight or digest* or diet or nutrition or malnutrition or deficien* or illness* or death* or morbidity or mortality or infection* or transmi* or sepsis* or enterocolitis* or cytomegalovirus* or HIV or immunologic or diarrh* or meningitis or urinary tract infection* or UTI or Expos* or Quality or Complication*).ti,ab,kf. | 29.087.092 |
| 10 | 8 and 9 | 34.472 |
| 11 | ((infant* or baby or babies or neonate* or new born* or newborn* or preterm or pre-term or child*) adj4 (Outcome* or impact* or affect* or influenc* or result* or consequenc* or caus* or issue* or threat* or effect* or benefit* or harm* or risk factor* or tolera* or growth* or development* or neurodevelop* or behavio* or weight or digest* or diet or nutrition or malnutrition or deficien* or illness* or death* or morbidity or mortality or infection* or transmi* or sepsis* or enterocolitis* or cytomegalovirus* or HIV or immunologic or diarrh* or meningitis or urinary tract infection* or UTI or Expos* or Quality or Complication*)).ti,ab,kf. | 920.955 |
| 12 | ((infant* or baby or babies or neonate* or new born* or newborn* or preterm or pre-term or child*).ti,ab,kf. or exp *Infant/) and exp *"growth, development and aging"/ | 206.215 |
| 13 | 10 or 11 or 12 | 1.046.892 |
| 14 | 7 and 13 | 12.008 |
| 15 | 14 not ((exp animal/ or nonhuman/) not exp human/) | 11.258 |
| 16 | limit 15 to "remove medline records" | 3.926 |

|  | **Search Strategy for EMCARE** | **Results** |
| --- | --- | --- |
| 1 | *breast milk/ or exp *Breast Feeding/ or exp *Lactation/ | 13.140 |
| 2 | (express* or pump* or extract* or collect* or treat* or pasteuri* or heat or thermal or composition or process* or microbial or antimicrobial or contaminat* or freez* or froze* or sonic* or UV or hygien* or save* or saving or stor* or keep* or conserv* or preserv* or Unpasteuri* or Steril* or microbiolog* or bacteria* or cool* or macronutrient*).ti,ab,kf. | 3.597.421 |
| 3 | 1 and 2 | 5.680 |
| 4 | ((breastmilk or ((breast or human or mother* or parent or parents) and milk) or breastfe* or breast feed* or breast fed or colostrum or lactat*) adj3 (express* or pump* or extract* or collect* or treat* or pasteuri* or heat or thermal or composition or process* or microbial or antimicrobial or contaminat* or freez* or froze* or sonic* or UV or hygien* or save* or saving or stor* or keep* or conserv* or preserv* or Unpasteuri* or Steril* or microbiolog* or bacteria* or cool* or macronutrient*)).ti,ab,kf. | 7.584 |
| 5 | Breast Milk Expression/ | 165 |
| 6 | hand express*.ti,ab,kf. | 106 |
| 7 | 3 or 4 or 5 or 6 | 10.841 |
| 8 | exp *Infant/ | 24.365 |
| 9 | (Outcome* or impact* or affect* or influenc* or result* or consequenc* or caus* or issue* or threat* or effect* or benefit* or harm* or risk factor* or tolera* or growth* or development* or neurodevelop* or behavio* or weight or digest* or diet or nutrition or malnutrition or deficien* or illness* or death* or morbidity or mortality or infection* or transmi* or sepsis* or enterocolitis* or cytomegalovirus* or HIV or immunologic or diarrh* or meningitis or urinary tract infection* or UTI or Expos* or Quality or Complication*).ti,ab,kf. | 6.281.167 |
| 10 | 8 and 9 | 18.821 |
| 11 | ((infant* or baby or babies or neonate* or new born* or newborn* or preterm or pre-term or child*) adj4 (Outcome* or impact* or affect* or influenc* or result* or consequenc* or caus* or issue* or threat* or effect* or benefit* or harm* or risk factor* or tolera* or growth* or development* or neurodevelop* or behavio* or weight or digest* or diet or nutrition or malnutrition or deficien* or illness* or death* or morbidity or mortality or infection* or transmi* or sepsis* or enterocolitis* or cytomegalovirus* or HIV or immunologic or diarrh* or meningitis or urinary tract infection* or UTI or Expos* or Quality or Complication*)).ti,ab,kf. | 320.864 |
| 12 | ((infant* or baby or babies or neonate* or new born* or newborn* or preterm or pre-term or child*).ti,ab,kf. or exp *Infant/) and exp *"growth, development and aging"/ | 50.322 |
| 13 | 10 or 11 or 12 | 354.261 |
| 14 | 7 and 13 | 4.099 |
| 15 | 14 not ((exp animal/ or nonhuman/) not exp human/) | 4.014 |

|  | **Search Strategy for EU Clinical Trials** | **Results** |
| --- | --- | --- |
| ● | ("breastmilk" OR (("breast" OR "human" OR "mother*" OR "parent" OR "parents") AND "milk") OR "breastfe*" OR "breast feed*" OR "breast fed" OR "colostrum" OR "lactat*") AND ("express*" OR "pump*" OR "extract*" OR "collect*" OR "treat*" OR "pasteuri*" OR "heat" OR "thermal" OR "composition" OR "process*" OR "microbial" OR "antimicrobial" OR "contaminat*" OR "freez*" OR "froze*" OR "sonic*" OR "UV" OR "hygien*" OR "save*" OR "saving" OR "stor*" OR "keep*" OR "conserv*" OR "preserv*" OR "Unpasteuri*" OR "Steril*" OR "microbiolog*" OR "bacteria*" OR "cool*" OR "macronutrient*") | 18 |

|  | **Search Strategy for Global Health** | **Results** |
| --- | --- | --- |
| 1 | ((breastmilk or ((breast or human or mother* or parent or parents) and milk) or breastfe* or breast feed* or breast fed or colostrum or lactat*) adj3 (express* or pump* or extract* or collect* or treat* or pasteuri* or heat or thermal or composition or process* or fortif* or microbial or antimicrobial or contaminat* or freez* or froze* or sonic* or UV or hygien* or save* or saving or stor* or keep* or conserv* or preserv* or Unpasteuri* or Steril* or microbiolog* or bacteria* or cool* or macronutrient*)).ti,ab,bt,sh. | 21.840 |
| 2 | hand express*.ti,ab,bt,sh. | 83 |
| 3 | 1 or 2 | 21.880 |
| 4 | ((infant* or baby or babies or neonat* or new born* or newborn* or preterm or pre-term or child*) adj4 (Outcome* or impact* or affect* or influenc* or result* or consequenc* or caus* or issue* or threat* or effect* or benefit* or harm* or risk factor* or tolera* or growth* or development* or neurodevelop* or behavio* or weight or digest* or diet or nutrition or malnutrition or deficien* or illness* or death* or morbidity or mortality or infection* or transmi* or sepsis* or enterocolitis* or cytomegalovirus* or HIV or immunologic or diarrh* or meningitis or urinary tract infection* or UTI or Expos* or Quality or Complication*)).ti,ab,bt,sh. | 372.406 |
| 5 | 3 and 4 | 7.346 |

|  | **Search Strategy for Global Index Medicus** | **Results** |
| --- | --- | --- |
| 1 | ( mh:("Breast Milk Expression")) | 52 |
| 2 | tw:(expressed breastmilk) | 30 |
| 3 | tw:(expressed breast milk) | 165 |
| 4 | tw:(breast milk pasteurization) | 42 |
|  | 1 OR 2 OR 3 OR 4 | 235 |

|  | **Search Strategy for Medline** | **Results** |
| --- | --- | --- |
| 1 | *Milk, Human/ or *Breast Feeding/ or exp *Lactation/ | 62.852 |
| 2 | (express* or pump* or extract* or collect* or treat* or pasteuri* or heat or thermal or composition or process* or microbial or antimicrobial or contaminat* or freez* or froze* or sonic* or UV or hygien* or save* or saving or stor* or keep* or conserv* or preserv* or Unpasteuri* or Steril* or microbiolog* or bacteria* or cool* or macronutrient*).ti,ab,kf. | 15.129.374 |
| 3 | 1 and 2 | 25.040 |
| 4 | ((breastmilk or ((breast or human or mother* or parent or parents) and milk) or breastfe* or breast feed* or breast fed or colostrum or lactat*) adj3 (express* or pump* or extract* or collect* or treat* or pasteuri* or heat or thermal or composition or process* or microbial or antimicrobial or contaminat* or freez* or froze* or sonic* or UV or hygien* or save* or saving or stor* or keep* or conserv* or preserv* or Unpasteuri* or Steril* or microbiolog* or bacteria* or cool* or macronutrient*)).ti,ab,kf. | 24.281 |
| 5 | Breast Milk Expression/ | 403 |
| 6 | hand express*.ti,ab,kf. | 459 |
| 7 | 3 or 4 or 5 or 6 | 41.195 |
| 8 | exp *Infant/ | 67.090 |
| 9 | (Outcome* or impact* or affect* or influenc* or result* or consequenc* or caus* or issue* or threat* or effect* or benefit* or harm* or risk factor* or tolera* or growth* or development* or neurodevelop* or behavio* or weight or digest* or diet or nutrition or malnutrition or deficien* or illness* or death* or morbidity or mortality or infection* or transmi* or sepsis* or enterocolitis* or cytomegalovirus* or HIV or immunologic or diarrh* or meningitis or urinary tract infection* or UTI or Expos* or Quality or Complication*).ti,ab,kf. | 23.608.974 |
| 10 | 8 and 9 | 48.946 |
| 11 | ((infant* or baby or babies or neonate* or new born* or newborn* or preterm or pre-term or child*) adj4 (Outcome* or impact* or affect* or influenc* or result* or consequenc* or caus* or issue* or threat* or effect* or benefit* or harm* or risk factor* or tolera* or growth* or development* or neurodevelop* or behavio* or weight or digest* or diet or nutrition or malnutrition or deficien* or illness* or death* or morbidity or mortality or infection* or transmi* or sepsis* or enterocolitis* or cytomegalovirus* or HIV or immunologic or diarrh* or meningitis or urinary tract infection* or UTI or Expos* or Quality or Complication*)).ti,ab,kf. | 706.458 |
| 12 | ((infant* or baby or babies or neonate* or new born* or newborn* or preterm or pre-term or child*).ti,ab,kf. or exp *Infant/) and exp *"Growth and Development"/ | 79.305 |
| 13 | 10 or 11 or 12 | 762.382 |
| 14 | 7 and 13 | 9.371 |
| 15 | 14 not (Animals/ not (Animals/ and Humans/)) | 9.059 |

|  | **Search Strategy for SCOPUS** | **Results** |
| --- | --- | --- |
| ● | ( TITLE-ABS-KEY ( ( "breastmilk" OR ( ( "breast" OR "human" OR "mother*" OR "parent" OR "parents" ) "milk" ) ) ) W/4 TITLE-ABS-KEY ( ( "express*" OR "pump*" OR "extract*" OR "collect*" OR "treat*" OR "pasteuri*" OR "heat" OR "thermal" OR "composition" OR "process*" OR "microbial" OR "antimicrobial" OR "contaminat*" OR "freez*" OR "froze*" OR "sonic*" OR "UV" OR "hygien*" OR "save*" OR "saving" OR "stor*" OR "keep*" OR "conserv*" OR "preserv*" OR "Unpasteuri*" OR "Steril*" OR "microbiolog*" OR "bacteria*" OR "cool*" OR "macronutrient*" ) ) W/4 TITLE-ABS-KEY ( ( "Outcome*" OR "impact*" OR "affect*" OR "influenc*" OR "result*" OR "consequenc*" OR "caus*" OR "issue*" OR "threat*" OR "effect*" OR "benefit*" OR "harm*" OR "risk factor*" OR "tolera*" OR "growth*" OR "development*" OR "neurodevelop*" OR "behavio*" OR "weight" OR "digest*" OR "diet" OR "nutrition" OR "malnutrition" OR "deficien*" OR "illness*" OR "death*" OR "morbidity" OR "mortality" OR "infection*" OR "transmi*" OR "sepsis*" OR "enterocolitis*" OR "cytomegalovirus*" OR "HIV" OR "immunologic" OR "diarrh*" OR "meningitis" OR "urinary tract infection*" OR "UTI" OR "Expos*" OR "Quality" OR "Complication*" ) ) W/4 TITLE-ABS-KEY ( ( "infant*" OR "baby" OR "babies" OR "neonate*" OR "new born*" OR "newborn*" OR "preterm" OR "pre-term" OR "child*" ) ) ) | 2.924 |

|  | **Search Strategy for ICTRP Registry** | **Results** |
| --- | --- | --- |
| ● | ("breastmilk" OR (("breast" OR "human" OR "mother*" OR "parent" OR "parents") AND "milk") OR "breastfe*" OR "breast feed*" OR "breast fed" OR "colostrum" OR "lactat*") AND ("express*" OR "pump*" OR "extract*" OR "collect*" OR "treat*" OR "pasteuri*" OR "heat" OR "thermal" OR "composition" OR "process*" OR "microbial" OR "antimicrobial" OR "contaminat*" OR "freez*" OR "froze*" OR "sonic*" OR "UV" OR "hygien*" OR "save*" OR "saving" OR "stor*" OR "keep*" OR "conserv*" OR "preserv*" OR "Unpasteuri*" OR "Steril*" OR "microbiolog*" OR "bacteria*" OR "cool*" OR "macronutrient*") | 1.315 |

|  | **Search Strategy for WoS** | **Results** |
| --- | --- | --- |
| ● | TS=(("breastmilk" OR (("breast" OR "human" OR "mother*" OR "parent" OR "parents") NEAR/3 "milk") OR "breastfe*" OR "breast feed*" OR "breast fed" OR "colostrum" OR "lactat*") NEAR/4 ("express*" OR "pump*" OR "extract*" OR "collect*" OR "treat*" OR "pasteuri*" OR "heat" OR "thermal" OR "composition" OR "process*" OR "microbial" OR "antimicrobial" OR "contaminat*" OR "freez*" OR "froze*" OR "sonic*" OR "UV" OR "hygien*" OR "save*" OR "saving" OR "stor*" OR "keep*" OR "conserv*" OR "preserv*" OR "Unpasteuri*" OR "Steril*" OR "microbiolog*" OR "bacteria*" OR "cool*" OR "macronutrient*") NEAR/4 ("Outcome*" OR "impact*" OR "affect*" OR "influenc*" OR "result*" OR "consequenc*" OR "caus*" OR "issue*" OR "threat*" OR "effect*" OR "benefit*" OR "harm*" OR "risk factor*" OR "tolera*" OR "growth*" OR "development*" OR "neurodevelop*" OR "behavio*" OR "weight" OR "digest*" OR "diet" OR "nutrition" OR "malnutrition" OR "deficien*" OR "illness*" OR "death*" OR "morbidity" OR "mortality" OR "infection*" OR "transmi*" OR "sepsis*" OR "enterocolitis*" OR "cytomegalovirus*" OR "HIV" OR "immunologic" OR "diarrh*" OR "meningitis" OR "urinary tract infection*" OR "UTI" OR "Expos*" OR "Quality" OR "Complication*") NEAR/4 ("infant*" OR "baby" OR "babies" OR "neonate*" OR "new born*" OR "newborn*" OR "preterm" OR "pre-term" OR "child*") ) | 709 |

**Supplemental Table 2: Quality Assessment of RCTs for both Methodology and Results and includes Risk of Bias**

**
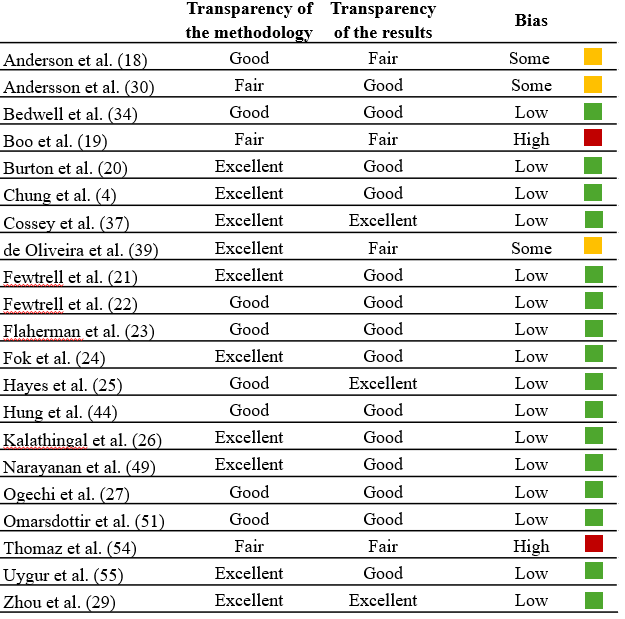
**

*RCTs – Randomized controlled trials.*

**Please note:** The Transparency of the Methodology and Results is grouped around the categorical classifications of Excellent - refers to the scores that fell within the fourth quartile, Good - refers to the third quartile, Fair - refers to the second quartile range, and Poor - refers to scores within the first quartile range. Regarding the Bias classification, we determinized Low bias to be the category of either Excellent or Good levels of transparency, and High bias to be the category of either Fair or Poor exclusively. If there was a combination of Excellent/Good and Fair/Poor the classification Some (yellow) was determined. The complete scores can be viewed in **Supplementary tables 3 and 4** where ‘Yes’ was scored 2, ‘I can't tell’ scored 1 and ‘No’ scored 0, with some categories not being applicable for some articles. Quartiles were determined from each of these articles using these scores.

**Supplemental Table 3: Detailed Data of Quality Assessment of RCTs methodology**

| **Study** | **Methodology** | | | | | | | | | | | | | |
| --- | --- | --- | --- | --- | --- | --- | --- | --- | --- | --- | --- | --- | --- | --- |
|  | Did the study address a clearly focused research question? | Are the inclusion criteria clearly described? | Was the method of randomization appropriate? | Was randomization sufficient to eliminate systematic bias? | Was the allocation sequence concealed from investigators and participants? | Were losses to follow-up and exclusions after randomization accounted for? | Were participants analyzed in the study groups to which they were randomized (intention-to-treat analysis)? | Was the study stopped early? If so, what was the reason? | Were the participants (mothers, caregivers) ‘blind’ to the intervention they were given? | Were the investigators ‘blind’ to the intervention they were giving to participants? | Were the people assessing/analyzing outcome/s ‘blinded’? | Were the study groups similar at the start of the randomized controlled trial? | Apart from the experimental intervention, did each study group receive the same level of care (that is, were they treated equally)? | Overall score |
| Anderson et al. (18) | 2 | 2 | 2 | 0 | 0 | 2 | 2 | 2 | 0 | 0 | 0 | 2 | 2 | 16/26 |
| Andersson et al. (30) | 2 | 2 | 0 | 0 | 0 |  | 0 | 2 | 0 | 0 | 0 | 1 | 2 | 9/24 |
| Bedwell et al. (34) | 2 | 2 | 2 | 2 | 0 | 2 | 2 | 2 | 0 | 0 | 0 | 2 | 2 | 18/26 |
| Boo et al. (19) | 2 | 2 | 0 | 0 | 0 | 0 | 0 | 2 | 0 | 0 | 0 | 2 | 1 | 9/26 |
| Burton et al. (20) | 2 | 2 | 2 | 2 | 2 | 2 | 2 | 2 | 0 | 0 | 0 | 2 | 2 | 20/26 |
| Chung et al. (4) | 2 | 2 | 2 | 2 | 2 | 2 | 2 | 2 | 2 | 2 | 0 | 2 | 2 | 24/26 |
| Cossey et al. (37) | 2 | 2 | 2 | 2 | 1 |  | 2 | 2 | 2 | 1 | 1 | 1 | 2 | 20/24 |
| de Oliveira et al. (39) | 2 | 2 | 2 | 2 | 2 | 2 | 2 | 2 | 2 | 2 | 2 | 2 | 2 | 26/26 |
| Fewtrell et al. (21) | 2 | 2 | 2 | 2 | 0 | 2 | 2 | 2 | 2 | 2 | 0 | 2 | 2 | 22/26 |
| Fewtrell et al. (22) | 2 | 2 | 2 | 2 | 0 | 0 | 2 | 2 | 0 | 0 | 1 | 1 | 2 | 16/26 |
| Flaherman et al. (23) | 2 | 2 | 2 | 2 | 0 | 2 | 2 | 2 | 0 | 0 | 0 | 2 | 2 | 18/26 |
| Fok et al. (24) | 2 | 2 | 2 | 2 | 2 | 2 | 2 | 2 | 2 | 2 | 2 | 2 | 2 | 26/26 |
| Hayes et al. (25) | 2 | 2 | 2 | 1 | 0 | 0 | 2 | 2 | 0 | 0 | 1 | 2 | 2 | 16/26 |
| Hung et al. (44) | 2 | 2 | 2 | 2 | 2 | 1 | 2 | 2 | 1 | 0 | 0 | 1 | 1 | 18/26 |
| Kalathingal et al. (26) | 2 | 2 | 2 | 2 | 2 | 2 | 2 | 2 | 2 | 2 | 2 | 2 | 2 | 26/26 |
| Narayanan et al. (49) | 2 | 2 | 2 | 2 | 2 | 2 | 2 | 2 | 0 | 0 | 0 | 2 | 2 | 20/26 |
| Ogechi et al. (27) | 2 | 2 | 2 | 2 | 0 |  | 2 | 2 | 0 | 0 | 0 | 2 | 2 | 16/24 |
| Omarsdottir et al. (51) | 2 | 2 | 2 | 2 | 2 | 0 | 0 | 2 | 0 | 0 | 0 | 2 | 2 | 16/26 |
| Thomaz et al. (54) | 2 | 2 |  |  |  | 0 |  | 2 | 0 | 0 | 0 | 2 | 1 | 9/18 |
| Uygur et al. (55) | 2 | 2 | 2 | 2 | 2 | 2 | 2 | 2 | 0 | 0 | 0 | 2 | 2 | 20/26 |
| Zhou et al. (29) | 2 | 2 | 2 | 2 | 2 | 0 | 2 | 2 | 2 | 2 | 1 | 2 | 1 | 22/26 |

**Note**: Empty cells stand for not applicable; 2 stands for ‘no’, 0 for ‘yes’. RCTs – Randomized controlled trials.

**Supplemental Table 4: Detailed Data of Quality Assessment of RCTs results**

| **Study** | **Results** | | | | | | | | | | |
| --- | --- | --- | --- | --- | --- | --- | --- | --- | --- | --- | --- |
|  | Was a power calculation undertaken? | Were the outcomes clearly specified? | Were the effects reported? | Were the results reported for each outcome in each study group at each follow-up interval? | Was there any missing or incomplete data?* | Was there a differential drop-out between the study groups that could affect the results?* | Were potential sources of bias identified? | Were appropriate statistical tests used? | Were p values reported? | Was the precision of the estimate of the intervention or treatment effect reported? Were confidence intervals (CIs) reported? | Overall score |
| Anderson et al. (18) | 0 | 2 | 0 | 2 | 0 | 2 | 0 | 2 | 2 | 0 | 10/20 |
| Andersson et al. (30) | 0 | 2 | 0 | 2 | 2 | 2 | 0 | 2 | 2 | 0 | 12/20 |
| Bedwell et al. (34) | 2 | 2 | 0 | 2 | 2 | 2 | 0 | 2 | 2 | 0 | 14/20 |
| Boo et al. (19) | 0 | 2 | 0 | 2 | 1 | 1 | 0 | 2 | 2 | 0 | 10/20 |
| Burton et al. (20) | 2 | 2 | 2 | 2 | 0 | 0 |  |  |  |  | 8/12 |
| Chung et al. (4) | 2 | 2 | 0 | 2 | 2 | 2 | 0 | 2 | 2 | 0 | 14/20 |
| Cossey et al. (37) | 0 | 2 | 0 | 2 | 2 | 2 | 0 | 2 | 2 | 2 | 14/20 |
| de Oliveira et al. (39) | 2 | 2 | 2 | 2 | 2 | 2 | 0 | 2 | 2 | 2 | 18/20 |
| Fewtrell et al. (21) | 2 | 2 | 0 | 2 | 0 | 0 | 0 | 2 | 2 | 0 | 10/20 |
| Fewtrell et al. (22) | 0 | 2 | 0 | 2 | 2 | 2 | 0 | 2 | 2 | 2 | 14/20 |
| Flaherman et al. (23) | 2 | 2 | 0 | 2 | 2 | 2 | 0 | 2 | 2 | 0 | 14/20 |
| Fok et al. (24) | 2 | 2 | 0 | 2 | 0 | 0 | 0 | 2 | 2 | 2 | 12/20 |
| Hayes et al. (25) | 2 | 2 | 2 | 2 | 2 | 2 | 0 | 2 | 2 | 2 | 18/20 |
| Hung et al. (44) | 0 | 2 | 2 | 2 | 2 | 2 | 0 | 2 | 2 | 0 | 14/20 |
| Kalathingal et al. (26) | 2 | 2 | 2 | 2 | 0 | 0 | 2 | 2 | 2 | 0 | 14/20 |
| Narayanan et al. (49) | 0 | 2 | 0 | 2 | 2 | 2 | 0 | 2 | 2 | 0 | 12/20 |
| Ogechi et al. (27) | 0 | 2 | 2 | 2 | 2 | 2 | 0 | 2 | 2 | 0 | 14/20 |
| Omarsdottir et al. (51) | 0 | 2 | 0 | 2 | 2 | 2 | 0 | 2 | 2 | 0 | 12/20 |
| Thomaz et al. (54) | 0 | 2 | 0 | 2 | 0 | 2 | 0 | 2 | 2 | 0 | 3/8 |
| Uygur et al. (55) | 2 | 2 | 0 | 2 | 2 | 2 | 0 | 2 | 2 | 0 | 14/20 |
| Zhou et al. (29) | 2 | 2 | 0 | 2 | 2 | 2 | 0 | 2 | 2 | 2 | 16/20 |

**Note**: Empty cells stand for not applicable; * 2 stands for ‘no’, 0 for ‘yes’ RCTs – Randomized controlled trials; CIs – confidence intervals

**Supplemental Table 5: Quality Assessment of Observational Studies for both Methodology and Results and includes Risk of Bias**


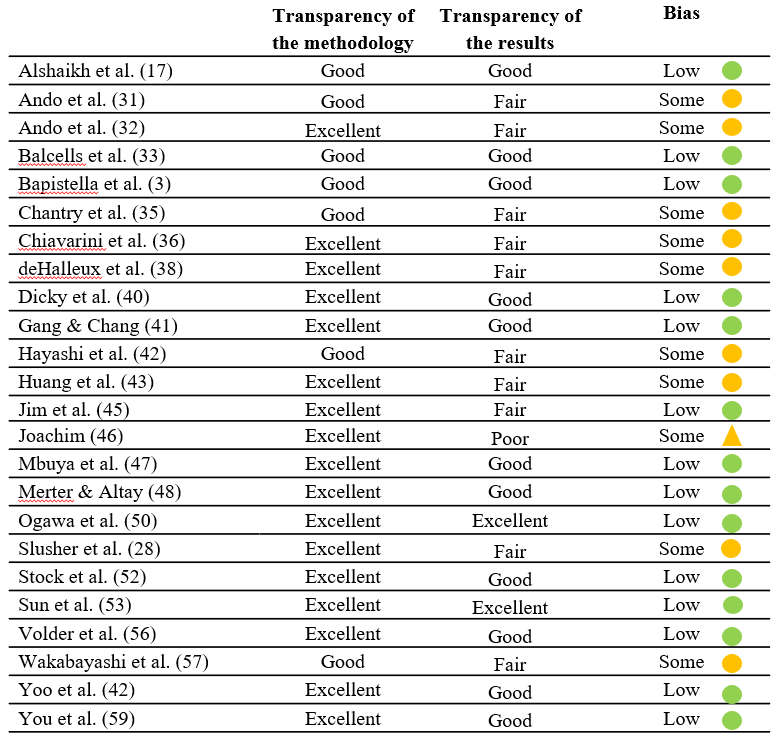


**Please note**: ○ Observational study; Δ On-going study. The Transparency of the Methodology and Results is grouped around the categorical classifications of Excellent - refers to the scores that fell within the fourth quartile, Good - refers to the third quartile, Fair - refers to the second quartile range, and Poor - refers to scores within the first quartile range regarding transparency. Regarding the Bias classification, we determinized Low bias to be the category of either Excellent or Good levels of transparency, and High bias to be the category of either Fair or Poor exclusively. If there was a combination of Excellent/Good and Fair/Poor the classification Some (yellow) was determined. The complete scores can be viewed in **Supplementary Tables 6 and 7** where ‘Yes’ was scored 2, ‘I can't tell’ scored 1 and ‘No’ scored 0, with some categories not being applicable for some articles. Quartiles were determined from each of these articles using these scores.

**Supplemental Table 6. Detailed Data of Quality Assessment of Observational Studies’ methodology**

|  | **Methodology** | | | | | | | | |  |
| --- | --- | --- | --- | --- | --- | --- | --- | --- | --- | --- |
| **Study** | Did the study address a clearly focused research question? | Are the inclusion criteria clearly described? | Are methods of follow up clearly described? | Are matching criteria clearly described? | Are variables identified and clearly described? | Is the sample relevant to the research goals? | | Sample recruitment: random | Are the measurement methods relevant to the research goals? | Overall score |
| Alshaikh et al. (17) | 2 | 2 | 0 |  | 2 | | 2 | 0 | 2 | 10/14 |
| Ando et al. (31) | 2 | 2 | 2 |  | 2 | | 1 | 0 | 0 | 9/14 |
| Ando et al. (32) | 2 | 2 | 2 |  | 2 | | 2 | 0 | 1 | 11/14 |
| Balcells et al. (33) | 2 | 2 | 0 | 0 | 2 | | 2 | 0 | 1 | 9/16 |
| Bapistella et al. (3) | 2 | 2 | 0 | 0 | 2 | | 2 | 0 | 2 | 10/16 |
| Chantry et al. (35) | 2 | 2 | 0 | 0 | 2 | | 2 | 0 | 2 | 10/16 |
| Chiavarini et al. (36) | 2 | 2 | 2 |  | 2 | | 2 | 0 | 2 | 12/14 |
| de Halleux et al. (38) | 2 | 2 | 2 | 2 | 2 | | 2 | 0 | 2 | 14/16 |
| Dicky et al. (40) | 2 | 2 | 2 | 2 | 2 | | 2 | 0 | 2 | 14/16 |
| Gang & Chang (41) | 2 | 2 | 2 |  | 2 | | 2 | 0 | 2 | 12/14 |
| Hayashi et al. (42) | 2 | 2 | 0 | 0 | 2 | | 2 | 0 | 2 | 10/16 |
| Huang et al. (43) | 2 | 2 | 2 | 1 | 2 | | 2 | 0 | 2 | 13/16 |
| Jim et al. (45) | 2 | 2 | 0 | 2 | 2 | | 2 | 0 | 2 | 12/16 |
| Joachim (46) | 2 | 2 | 1 | 1 | 2 | | 2 | 0 | 2 | 12/16 |
| Mbuya et al. (47) | 2 | 2 |  |  | 2 | | 2 | 0 | 2 | 10/12 |
| Merter & Altay (48) | 2 | 2 | 2 | 2 | 2 | | 2 | 0 | 2 | 14/16 |
| Ogawa et al. (50) | 2 | 2 | 2 | 2 | 2 | | 2 | 0 | 2 | 14/16 |
| Slusher et al. (28) | 2 | 2 | 2 |  | 2 | | 2 | 0 | 2 | 12/14 |
| Stock et al. (52) | 2 | 2 | 2 | 2 | 2 | | 2 | 0 | 2 | 14/16 |
| Sun et al. (53) | 2 | 2 | 2 | 1 | 2 | | 2 | 0 | 2 | 13/16 |
| Volder et al. (56) | 2 | 2 | 2 |  | 2 | | 2 | 0 | 2 | 12/14 |
| Wakabayashi et al. (57) | 2 | 2 | 0 | 0 | 2 | | 2 | 0 | 2 | 10/16 |
| Yoo et al. (58) | 2 | 2 | 2 | 1 | 2 | | 2 | 0 | 2 | 13/16 |
| You et al. (59) | 2 | 2 | 2 |  | 2 | | 2 | 0 | 2 | 12/14 |

**Note**: Empty cells stand for not applicable; * 2 stands for ‘no’, 0 for ‘yes’

**Supplemental Table 7: Detailed Data of Quality Assessment of Observational Studies’ results**

| **Study** | **Results** | | | | | | | | | | | | | **Overall score** |  |
| --- | --- | --- | --- | --- | --- | --- | --- | --- | --- | --- | --- | --- | --- | --- | --- |
|  | Was a sample power calculation undertaken? | Were the outcomes clearly specified? | Were the effects reported? | Were the results reported for each outcome in each study group at each stage (if applicable)? | Was there any missing or incomplete data? * | Were the missing data addressed (explained, estimated)? | Was there a differential drop-out between the measurements (if applicable)?* | Were potential sources of bias identified? | Were appropriate statistical tests used? | Were p values reported? | Was the precision of the estimate of the intervention or treatment effect reported? Were confidence intervals (CIs) reported? | Is the participants characteristics provided? | Cohort studies: Was the follow-uip time summarized? |  | Was the confounding addressed? |
| Alshaikh et al. (17) | 0 | 2 | 2 | 2 | 2 |  | 1 | 0 | 2 | 2 | 2 | 2 | 0 | 17/24 | 1 |
| Ando et al. (31) | 0 | 2 | 0 | 0 | 1 | 1 | 2 | 0 | 2 | 0 | 0 | 0 | 0 | 8/26 | 0 |
| Ando et al. (32) | 0 | 2 | 0 | 2 | 1 | 1 | 2 | 0 | 1 | 0 | 0 | 0 | 0 | 9/26 | 0 |
| Balcells et al. (33) | 0 | 2 | 0 | 0 | 2 |  | 2 | 0 | 1 | 2 | 2 | 2 | 0 | 13/24 | 1 |
| Bapistella et al. (3) | 0 | 2 | 0 | 2 | 1 | 1 | 2 | 0 | 2 | 2 | 0 | 2 | 0 | 14/26 | 1 |
| Chantry et al. (35) | 0 | 2 | 0 | 0 | 2 |  | 2 | 0 | 2 | 2 | 0 | 2 | 0 | 12/24 | 1 |
| Chiavarini et al. (36) | 0 | 2 | 0 | 2 | 2 |  | 2 |  | 1 | 0 | 0 | 2 | 0 | 11/22 | 0 |
| de Halleux et al. (38) | 0 | 2 | 0 | 2 | 0 | 2 | 2 | 0 | 1 | 0 | 2 | 2 | 0 | 13/26 | 1 |
| Dicky et al. (40) | 2 | 2 | 0 | 2 | 2 |  | 2 | 2 | 2 | 2 | 2 | 0 | 0 | 18/24 | 2 |
| Gang & Chang (41) | 0 | 2 | 0 | 2 | 2 |  | 2 | 0 | 2 | 2 | 0 | 2 |  | 14/22 | 1 |
| Hayashi et al. (42) | 0 | 2 | 0 | 2 | 2 |  | 2 | 0 | 2 | 2 | 0 | 0 | 0 | 12/24 | 1 |
| Huang et al. (43) | 0 | 2 | 2 | 0 | 2 |  | 2 | 0 | 2 | 2 | 0 | 0 | 0 | 12/24 | 2 |
| Jim et al. (45) | 0 | 2 | 0 | 0 | 2 |  | 2 | 0 | 2 | 2 | 0 | 2 | 0 | 12/24 | 2 |
| Joachim (46) | 0 | 2 | 0 | 0 | 0 | 0 |  | 0 |  |  |  |  |  | 2/14 | 1 |
| Mbuya et al. (47) | 0 | 2 | 0 | 0 | 2 |  | 2 | 0 | 2 | 2 | 0 | 2 |  | 12/22 | 2 |
| Merter & Altay (48) | 0 | 2 | 2 | 0 | 2 |  | 2 | 0 | 2 | 2 | 0 | 2 | 0 | 14/24 | 2 |
| Ogawa et al. (50) | 0 | 2 | 2 | 2 | 2 |  | 2 | 0 | 2 | 2 | 2 | 2 | 2 | 20/24 | 1 |
| Slusher et al. (28) | 0 | 2 | 0 | 0 | 2 |  | 2 | 0 | 2 | 0 | 0 | 2 | 0 | 10/24 | 1 |
| Stock et al. (52) | 0 | 2 | 0 | 2 | 2 |  | 2 | 0 | 2 | 2 | 0 | 2 |  | 14/22 | 2 |
| Sun et al. (53) | 0 | 2 | 2 | 2 | 0 | 2 | 2 | 0 | 2 | 2 | 2 | 2 | 2 | 20/26 | 2 |
| Volder et al. (56) | 0 | 2 | 2 | 2 | 0 | 2 | 2 | 0 | 2 | 2 | 2 | 2 |  | 18/24 | 1 |
| Wakabayashi et al. (57) | 0 | 2 | 0 | 2 | 2 |  | 2 | 0 | 1 | 2 | 0 | 0 | 0 | 11/24 | 2 |
| Yoo et al. (58) | 0 | 2 | 0 | 0 | 2 |  | 2 | 0 | 2 | 2 | 2 | 2 |  | 14/22 | 2 |
| You et al. (59) | 0 | 2 | 0 | 2 | 2 |  | 2 | 0 | 2 | 2 | 0 | 2 |  | 14/22 | 2 |

**Note**: Empty cells stand for not applicable; * 2 stands for ‘no’, 0 for ‘yes’. Cis – confidence intervals.

**Supplemental Table 8: The certainty of evidence grading**

| **Intervention** | **Outcome under consideration** | **Relative importance of the outcome (1-9)** | **Studies under consideration** | **Design** | **Risk of Bias** | **Inconsistency** | **Indirectness** | **Imprecision** | **Other (Publication Bias)** | **Certainty (overall score)** | |
| --- | --- | --- | --- | --- | --- | --- | --- | --- | --- | --- | --- |
| Hindmilk | Growth | 9 | Alshaikh et al. (17); Ogechi et al. (27); Slusher et al. (28) | 1 RCT, 2 Obs | Serious | Serious | Not serious | Serious | Not at all serious | | Low |
|  | Nutrient deficiencies | 6 | Slusher et al. (28) | 1 Obs | Not at all serious | Not applicable (1 study only) | Serious | Serious | Not at all serious | | Very Low |
| Pumping method or regimen | Breastfeeding | 9 | Anderson et al. (18); Fewtrell et al. (21); Flaherman et al. (23); Fok et al. (24); Hayes et al. (25); Kalathingal et al. (26); Zhou et al. (29) | 8 RCT | Not really serious | Very serious | Not serious | Not at all serious | Not at all serious | | High |
|  | Morbidity | 9 | Fewtrell et al. (21) | 1 RCT | Not at all serious | Not applicable (1 study only) | Not serious | Not serious | Not at all serious | | High |
|  | Growth | 9 | Fok et al. (24); Kalathingal et al. (26) | 2 RCT | Not at all serious | Serious | Not serious | Not serious | Not at all serious | | High |
| Freeze-thawing | CMV infection | 8 | Chiavarini et al. (36); Hayashi et al. (42); Jim et al. (45); Ogawa et al. (50); Omarsdottir (51); Volder et al. (56); Wakabayashi et al. (57); You et al. (59) | 1 RCT, 7 Obs | Not really serious | Serious | Not serious | Serious | Not at all serious | | Low |
|  | Other infections | 9 | Omarsdottir (51); Sun et al. (53); You et al. (59) | 1 RCT, 2 Obs | Not at all serious | Serious | Not serious | Serious | Not at all serious | | Low |
|  | Retroviral infection | 9 | Ando et al. (31); Ando et al. (32) | 2 Obs | Not at all serious | Not really serious | Not serious | Serious | Not at all serious | | Low |
|  | Growth | 9 | Omarsdottir (51); Sun et al. (53); Wakabayashi et al. (57) | 1 RCT, 2 Obs | Not at all serious | Serious | Not serious | Serious | Not at all serious | | Low |
|  | Morbidity | 9 | Omarsdottir (51); Sun et al. (53); You et al. (59) | 1 RCT, 2 Obs | Not at all serious | Not really serious | Not at all serious | Serious | Not at all serious | | Moderate |
|  | Mortality | 9 | Omarsdottir (51); Sun et al. (53) | 1 RCT, 1 Obs | Not at all serious | Not really serious | Not at all serious | Serious | Not at all serious | | Moderate |
|  | Feeding tolerance | 8 | Omarsdottir (51); Sun et al. (53); You et al. (59) | 1 RCT, 2 Obs | Not at all serious | Serious | Not serious | Serious | Not at all serious | | Low |
| Heat treatment | Growth | 9 | Andersson et al. (30); Chantry et al. (35); Cossey et al. (37); Dicky et al. (40); Huang et al. (43); de Halleux et al. (38); Joachim (46); Mbuya & Altay (47); Yoo et al. (58) | 2 RCT, 7 Obs | Not really serious | Not really serious | Not serious | Not serious | Not at all serious | | Moderate |
|  | Mortality | 9 | Chung et al. (4); Cossey et al. (37); Dicky et al. (40); Huang et al. (43); Stock et al. (52); Yoo et al. (58) | 3 RCT, 3 Obs | Not at all serious | Not really serious | Not at all serious | Not serious | Not at all serious | | Moderate |
|  | Morbidity | 9 | Bapistella et al (3); Chung et al. (4); Cossey et al. (37); Dicky et al. (40); Huang et al. (43); Stock et al. (52); Yoo et al. (58) | 3 RCT, 4 Obs | Not at all serious | Not really serious | Not serious | Not serious | Not at all serious | | Moderate |
|  | Feeding tolerance | 8 | Chung et al. (4); Cossey et al. (37); de Oliveira (39); Dicky et al. (40); Huang et al. (43); Joachim (46); Stock et al. (52); Yoo et al. (58) | 4 RCT, 4 Obs | Not really serious | Not really serious | Not serious | Not at all serious | Not at all serious | | Moderate |
|  | Adverse events | 9 | Huang et al. (43) | 1 Obs | Not at all serious | Not applicable (1 study only) | Not at all serious | Serious | Not at all serious | | Very Low |
|  | CMV infection | 8 | Bapistella et al. (3); Chung et al. (4); Gang & Chang (41); Stock et al. (52); Yoo et al. (58) | 1 RCT, 4 Obs | Not at all serious | Not really serious | Not serious | Not serious | Not at all serious | | Moderate |
|  | Other infections | 9 | Chung et al. (4); Chantry et al. (35); Cossey et al. (37); Dicky et al. (40); Huang et al. (43); Narayanan et al. (49); Stock et al. (52); Yoo et al. (58) | 3 RCT, 4 Obs | Not at all serious | Not really serious | Serious | Not at all serious | Not at all serious | | Moderate |
| Warming method | Growth | 9 | Bedwell (34); Uygur et al (55) | 2 RCT | Not at all serious | Very serious | Not serious | Serious | Not at all serious | | Moderate |
|  | Morbidity | 9 | Uygur et al (55) | 1 RCT | Not at all serious | Not applicable (1 study only) | Serious | Serious | Very serious | | Low |

**Supplemental Figure 1 EVIDENCE GAP MAP**

**
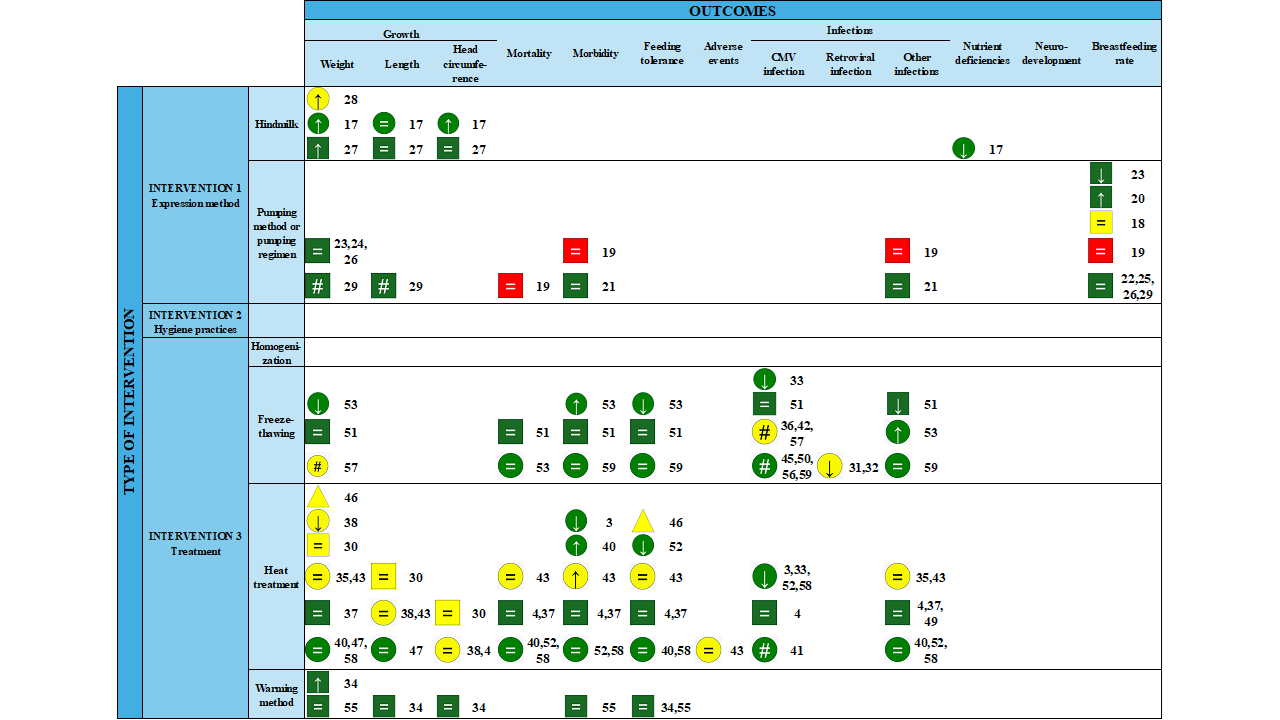
**

High quality RCT; Moderate quality RCT; Low quality RCT; High quality observational; Moderate quality observational; Ongoing study;

**↑** increased by intervention; = unaffected by intervention; ↓ decreased by intervention; # descriptive study

Each icon represents an outcome reported in an individual study. The numbers correspond to the reference list of the selected full-text articles. Please note there is no evidence for the intervention associated with hygiene. Morbidity includes BPD, NEC, ROP, IVH/PVL. Feeding tolerance was defined by duration of parenteral nutrition, time to full enteral feeding, or predefined feeding intolerance score. Adverse events were defined as an undesired effect of the intervention under evaluation. Other infections included bacterial infections, fungal infections, viral infections other than Cytomegalovirus (CMV) and retroviruses. Neurodevelopment was defined by a standardised neurodevelopment assessment, performed at any age.
